# Trends in incidence, mortality and disability-adjusted life years of colorectal cancer in East Asia (1990–2021): An analysis of the Global Burden of Disease study 2021

**DOI:** 10.1371/journal.pone.0334229

**Published:** 2025-10-08

**Authors:** Yuanyi Cao, Liyuan Yang, Luhan Fan, Xinyao Liu, Chao Yang, Zhengxing Xu, Qingbi Zhang, Junhui Zhang

**Affiliations:** 1 Department of Epidemiology and Health Statistics, School of Public Health, Southwest Medical University, Luzhou, Sichuan, China; 2 Center for Preventive Care and Health Examination, The Affiliated Traditional Chinese Medicine Hospital, Southwest Medical University, Luzhou, Sichuan, China; 3 Department of Occupational and Environmental Health, School of Public Health, Southwest Medical University, Luzhou, Sichuan, China; National Center for Chronic and Noncommunicable Disease Control and Prevention, Chinese Center for Disease Control and Prevention, CHINA

## Abstract

**Background:**

Colorectal cancer (CRC) poses a significant health challenge in East Asia. This study examined long-term trends in CRC incidence, mortality and disability-adjusted life years (DALYs) and their key drivers across five East Asian countries (China, Japan, South Korea, North Korea and Mongolia) from 1990–2021.

**Methods:**

Using Global Burden of Disease Study 2021 data, we assessed temporal trends using joinpoint regression, age/period/birth cohort effects through age-period-cohort analysis, and key drivers via decomposition analysis; we also evaluated associations between CRC burden and the Socio-Demographic Index (SDI).

**Results:**

Between 1990 and 2021, descriptive analysis showed CRC age-standardised incidence rates (ASIRs) increased in all East Asian countries, while age-standardised mortality rates (ASMRs) and age-standardised DALY rates (ASDRs) generally declined, although 95% uncertainty intervals (UIs) overlapped in some countries. Joinpoint regression revealed overall increasing ASIR trends, with South Korea showing the largest average annual percentage changes (AAPCs) (males 2.18%, females 1.41%). ASMRs and ASDRs generally decreased, particularly among females; however, Mongolian males experienced slight increases (ASMR AAPC = 0.50%; ASDR AAPC = 0.44%). Relative risks for CRC incidence, mortality and DALYs increased with age, peaking in older groups. Period effects increased approximately linearly, while cohort effects decreased for younger generations. Decomposition analysis showed ageing and population growth were the main contributors to increased CRC burden. SDI showed an overall positive association with CRC burden, with heterogeneity by country and sex.

**Conclusions:**

Joinpoint analysis (1990–2021) demonstrated increases in CRC incidence (predominantly males) and declines in mortality/DALY rates (especially females) across most East Asian countries; however, substantial changes (non-overlapping UIs) were limited to few groups, warranting cautious interpretation. Despite progress, the persistent overall burden remains substantial, mainly driven by ageing and population growth. These findings underscore the need for country- and sex-specific prevention, screening, and lifestyle strategies.

## Introduction

Colorectal cancer (CRC) is the third most common malignancy and the second leading cause of cancer-related death worldwide, with an estimated 1.9 million new cases and 0.9 million deaths in 2022 [1]. These numbers are projected to rise to 3.2 million cases and 1.6 million deaths by 2040 [2]. While early screening and treatment in high-income countries have stabilised the burden [3,4], East Asia has seen a steady increase in CRC incidence and mortality [5,6], now accounting for over 25% of global CRC cases and deaths [5].

Westernised diets (characterised by high fat, sugar, salt, and processed food intake and low dietary fibre consumption) [7], low physical activity and urbanisation have contributed substantially to this increase in CRC burden specifically in East Asia [6,8]. Despite shared exposure to risk factors, East Asian countries exhibit marked disparities in socioeconomic development, demographic structure, and screening infrastructure. Japan and South Korea, as high-income countries [9], benefit from mature national screening programmes predominantly utilising fecal immunochemical testing (FIT), supporting high participation rates [10,11]. Nevertheless, both face growing challenges in CRC prevention and control due to their rapidly ageing populations [12]. China and Mongolia, as middle-income economies, have experienced rapid urbanisation and dietary shifts [13,14]. Notably, Mongolia is the only country among them that has not yet entered an ageing society, thus presenting a stark contrast in population structure [15]. Meanwhile, China has implemented stepwise CRC screening programmes across rural and urban regions [16], while Mongolia has initiated a national screening programme utilising fecal occult blood tests (FOBTs) [13]. North Korea, a low-income country, constrained by scarce medical resources, lacks functional population-level screening [17]. Thus, comparing epidemiological trends across countries not only reveals the varied burden of CRC in East Asia but also provides invaluable insights for formulating targeted strategies under diverse socioeconomic, demographic, and screening contexts. Furthermore, the United States, as a developed country and a typical representative of the Western lifestyle, has successfully reduced its CRC burden through early screening [18], promotion of healthy diets and encouragement of physical activity [19]. Comparing the CRC trends in the five East Asian countries with those in the United States facilitates the identification of key gaps in CRC prevention and control in East Asia, providing a reference for more effective CRC prevention and control strategies in these countries.

Previous studies have documented regional variations in CRC trends globally [5], particularly between East Asia and the United States [1,4], attributable to factors such as national health policies, lifestyle changes, and screening rates [20]. However, most existing research has focused on individual East Asian countries, without adequately addressing the region’s diverse socioeconomic contexts and long-term dynamics [21,22]. These limitations hinder the development of region-specific prevention strategies, as CRC burden in East Asia is shaped by both shared risk factors and country-specific challenges.

This study aims to address this critical knowledge gap by comprehensively analysing trends in CRC incidence, mortality and DALYs in China, Japan, South Korea, North Korea and Mongolia from 1990 to 2021 using population-level data from the Global Burden of Disease (GBD) study, with a key focus on identifying the distinct contributions of ageing, population growth, and epidemiological changes through decomposition analysis. Furthermore, given that East Asian countries represent diverse stages of socio-economic development, this study also examines the association between CRC burden and the socio-demographic index (SDI). By comparing these findings with those of the United States and globally, this study provides crucial evidence to inform the development of targeted public health policies and CRC screening strategies in East Asia, thereby contributing substantially to global colorectal cancer prevention and control.

## Materials and methods

### Study setting

Five East Asian countries (China, Japan, South Korea, North Korea, and Mongolia) were included to capture the diversity of socioeconomic development stages, healthcare systems, and demographic profiles within the region [12]. The United States was selected as a comparator due to its developed healthcare system, well-established colorectal cancer prevention and screening programmes, and its role as a typical representative of the Western lifestyle [19]. Additionally, global data were incorporated to provide broader context. This selection facilitates a comprehensive comparison of colorectal cancer trends and enables the identification of region-specific challenges and opportunities.

### Data sources

Data for this study were obtained from the GBD 2021 database via the Global Health Data Exchange results tool (https://vizhub.healthdata.org/gbd-results/) [23]. The GBD, a comprehensive global health assessment project led by the Institute for Health Metrics and Evaluation at the University of Washington, aims to collect, evaluate and disseminate global health data to analyse the burden of diseases, injuries and risk factors worldwide. The GBD database is a regularly updated resource, offering data on incidence, mortality and DALYs stratified by country, year, sex and age [24].

The GBD 2021 estimates primarily rely on the Disease Modelling Meta-Regression version 2.1 (DisMod-MR 2.1), a Bayesian meta-regression framework that integrates heterogeneous data sources and addresses missing data through hierarchical modelling [25]. This approach is particularly important for generating robust estimates in regions with limited primary data, such as North Korea [26]. All GBD-derived metrics include 95% uncertainty intervals (UIs), calculated as the 2.5th and 97.5th percentiles of 500 draws from the posterior distribution [25].

For this investigation, we extracted the numbers and age-standardised rates (ASRs) of CRC incidence, mortality, and DALYs, along with their 95% UIs, for China, Japan, South Korea, North Korea and Mongolia over the period 1990–2021. We also obtained population data and annual values of the SDI, a composite measure that integrates per capita gross domestic product (GDP), average educational attainment for individuals aged 15 years and older, and the total fertility rate under age 25 [27]. SDI provides a standardised metric for assessing overall socio-economic development. Data were further categorised by sex (male/female) and age (17 groups, 15–19–95 + years, 5-year intervals) and compared with data from the global population and the United States.

### Statistical analysis

#### Joinpoint regression model.

Joinpoint regression was used to evaluate temporal trends in CRC age-standardised incidence rate (ASIR), age-standardised mortality rate (ASMR) and age-standardised DALY rate (ASDR) in the specified geographic region from 1990 to 2021. This method segments long-term trends into multiple periods, assessing and optimising the fit within each segment. As the data were not normally distributed, a log-linear model was applied. The location and number of joinpoints were determined using the grid search method, and statistical significance was assessed via Monte Carlo permutation tests [28]. To balance model complexity with statistical stability, a maximum of five joinpoints was permitted, given the 32-year study period. From this analysis, the annual percent change (APC) and its associated 95% confidence interval (CI) as well as the average annual percent change (AAPC), were calculated [29]. Where the APC and AAPC were concordant, no inflection points were identified. A trend was considered statistically significant when the 95% CI for the APC or AAPC did not include 0. Specifically, for CRC incidence, mortality or DALYs, an increasing trend was indicated if the APC or AAPC value and its 95% CI were both greater than 0; a decreasing trend was indicated if the value and its 95% CI were both less than 0; and a stable trend was indicated when the 95% CI included 0.

#### Age-period-cohort model.

To investigate the independent effects of age, period and birth cohort on CRC incidence, mortality and DALYs in the five East Asian countries, age-period-cohort analysis was performed. Age-period-cohort model is a statistical technique based on Poisson regression that is designed to assess disease trends across distinct age groups, time periods, and birth cohorts [30]. For the purposes of this study, age (e.g., 15–19, 20–24, 25–29,..., 90–94, 95+), observation periods (e.g., 1992–1996, 1997–2001,..., 2017–2021) and birth cohorts (e.g., 1897–1901, 1902–1906,..., 2002–2006) were grouped into 5-year intervals to estimate their net effects on CRC incidence, mortality and DALY trends.

Owing to inherent collinearity among age, period and birth cohort in the age-period-cohort model (where period minus age equals cohort), we used the intrinsic estimator (IE) method proposed by Fu et al [31,32]. This method mitigates the interdependence of these variables, enabling a more accurate analysis of their individual effects. The age-period-cohort model is expressed as follows:


$$\text{ln}\left(R_{ijk}\right)=\mu +\alpha_{i}+\beta_{j}+\gamma_{k}+\varepsilon_{ijk}$$

where $R_{ijk}$ denotes the CRC incidence, mortality and DALY rates in the *i*-th age group, *j*-th period and *k*-th birth cohort (expressed per 100,000 population); μ represents the intercept; $\alpha_{i}$, $\beta_{j}$ and $\gamma_{k}$ denote the regression coefficients for age, period and birth cohort effects, respectively. To facilitate interpretation in terms of relative risk (RR), the model parameters (α, β and γ) were exponentiated [33]. The resulting RRs represent the relative risk of CRC incidence, mortality, or DALYs for a specific age group, period or birth cohort relative to the reference group defined as the average level across all groups of the same type within each country and region. An RR value exceeding 1 signifies an elevated risk compared to the reference group, whereas an RR value below 1 signifies a reduced risk. An RR of 1 indicates no difference in risk compared with the reference group.

#### Decomposition analysis.

To analyse the factors driving changes in the numbers of CRC incident cases, deaths, and DALYs in the five East Asian countries between 1990 and 2021, we performed a decomposition analysis using the method proposed by Das Gupta [34–37]. This method allows for the partitioning of observed changes into three distinct components: ageing (reflecting the impact of changes in age structure on CRC burden), population growth (reflecting the impact of changes in total population size on CRC burden) and epidemiological changes (reflecting the impact of changes in age-specific rates on CRC burden). The epidemiological change component captures variations in rates that cannot be explained via demographic shifts (namely, population growth and ageing), and thus encompasses the combined effects of lifestyle, healthcare, prevention strategies, treatment, and other non-demographic determinants influencing CRC incidence, mortality, and DALYs. The basic formula is as follows (i.e., incidence):


$${Incidence}_{ay,\;py,\;ey}=\sum_{i=\text{1}}^{\text{1}\text{7}}\left(a_{i,y}\times p_{y}\times e_{i,y}\right)$$

where i represents the age group index (1–17), corresponding sequentially to the age groups: 1= 0–4 years, 2 = 5–9 years, …, 17 = 95+ years; and y denotes the study year (1990 or 2021). ${Incidence}_{ay,\;py,\;ey}$ represents the total number of CRC cases in year y, calculated based on the age structure, population size, and CRC incidence rate for that year; $a_{i,y}$ denotes the proportion of population in age group *i* in year y; $p_{y}$ is the total population in year y; and $e_{i,y}$ represents the CRC incidence rate in age group *i* in year y.

To quantify the independent contributions of ageing, population growth, and epidemiological changes, we set 1990 as the baseline year and 2021 as the target year, and constructed three counterfactual scenarios. In each scenario, one factor was updated to its 2021 level with the other factors held constant at their 1990 levels. By calculating the difference in the number of CRC cases between each scenario and the baseline values, the preliminary effect of each factor was estimated. Potential interactions between factors were accounted for using the Das Gupta method, which distributes the interaction effects among the factors to ensure that the sum of their independent contributions exactly equals the total observed change between the target and baseline years. Finally, the relative percentage contribution of each factor was quantified as the proportion of its effect to the total change. The same decomposition methodology was applied to analyse deaths and DALYs.

#### Association between SDI and CRC burden.

To assess the association between socioeconomic development and CRC burden, we calculated Spearman’s rank correlation coefficients (*r*) between annual SDI values and the ASRs of incidence, mortality, and DALYs for each of the Asian countries examined, the Asian regional aggregate, the United States, and globally from 1990 to 2021. The coefficient *r* ranges from −1 to +1, with absolute values closer to 1 indicating stronger associations [38]. Additionally, to explore potential non-linear relationships between CRC burden metrics (ASIR, ASMR, ASDR) and SDI, we employed Locally Estimated Scatterplot Smoothing (LOESS) regression, which effectively models the data through a non-parametric smoothing technique [39,40].

Two-sided statistical tests were conducted with a significance level of 0.05. Joinpoint regression was performed using the Joinpoint Regression Programme (version 5.0.2) from the National Cancer Institute. Age-period-cohort analysis, decomposition analysis, Spearman’s rank correlation, and LOESS regression were conducted using R software (versions 3.6.0 and 4.3.2).

## Results

### Description analysis of colorectal cancer burden

In 1990, there were 469.76 thousand global CRC diagnoses in males and 446.82 thousand in females, corresponding to ASIRs of 27.31 per 100,000 and 21.41 per 100,000, respectively. Among East Asian countries, Japan reported the highest ASIRs (55.97 per 100,000 for males and 63.96 per 100,000 for females), followed by South Korea, China, North Korea and Mongolia. By 2021, the global CRC ASIR had increased by 16.92% in males but decreased by 5.79% in females. All East Asian countries experienced increases in ASIRs for both sexes; notably, non-overlapping 95% UIs between 1990 and 2021 were primarily observed only among males in China, Japan, and South Korea. South Korea demonstrated the most substantial increase, with a rise of 96.26% in males. By 2021, the ranking of East Asian countries by ASIR remained the same as in 1990 for both sexes, with Japan’s ASIR being 5.47 times that of Mongolia (the lowest) for males and 4.03 times for females. Remarkably, the incidence gap between East Asia and the United States narrowed considerably, from 6.68-fold in 1990 to 3.84-fold in 2021 (Table 1).

**Table 1:** The cases and age-standardised rates (per 100,00 population) of CRC incidence, mortality and DALYs by sex in East Asian countries, the United States, and globally in 1990 and 2021.

Countries	Male					Female				
	1990		2021		Change in rates, %	1990		2021		Change in rates, %
	Cases No. × 10^3^ (95% UI)	ASR^*^ No. (95% UI)	Cases No. × 10^3^ (95% UI)	ASR^*^ No. (95% UI)		Cases No. × 10^3^ (95% UI)	ASR^*^ No. (95% UI)	Cases No. × 10^3^ (95% UI)	ASR^*^ No. (95% UI)	
**Incidence**										
China	88.37 (69.95, 107.47)	22.31 (17.92, 26.82)	419.01 (319.83, 541.67)	42.24 (32.56, 54.26)	89.33	70.02 (55.49, 85.56)	16.43 (13.18, 19.95)	239.31 (181.75, 305.84)	21.87 (16.58, 27.92)	33.11
Japan	41.17 (39.70, 42.59)	55.97 (53.67, 57.98)	98.95 (90.77, 104.60)	63.96 (59.31, 67.26)	14.28	31.92 (29.13, 33.52)	33.42 (30.55, 35.07)	72.09 (56.59, 81.79)	35.30 (30.02, 38.58)	5.63
South Korea	2.94 (2.43, 3.60)	24.84 (20.77, 30.31)	20.19 (16.14, 24.50)	48.75 (38.47, 59.21)	96.26	2.70 (2.20, 3.20)	15.98 (12.89, 19.11)	12.95 (9.76, 16.63)	24.93 (18.94, 31.82)	56.01
North Korea	1.11 (0.79, 1.61)	16.84 (12.28, 23.72)	2.75 (1.86, 4.33)	19.00 (12.95, 29.46)	12.83	1.14 (0.79, 1.74)	11.76 (8.27, 17.73)	2.40 (1.48, 3.79)	12.62 (7.77, 19.89)	7.31
Mongolia	0.04 (0.03, 0.06)	8.60 (6.10, 11.92)	0.12 (0.08, 0.17)	11.69 (8.11, 15.82)	35.93	0.05 (0.03, 0.06)	8.07 (5.78, 10.98)	0.12 (0.08, 0.15)	8.77 (6.19, 11.52)	8.67
United States	77.14 (73.65, 79.79)	57.47 (54.60, 59.50)	115.51 (108.86, 120.78)	44.91 (42.32, 46.88)	−21.85	75.18 (68.02, 79.48)	39.87 (36.56, 41.93)	98.60 (88.10, 104.98)	32.16 (29.28, 34.00)	−19.34
Global	469.76 (445.31, 492.30)	27.31 (25.89, 28.51)	1263.46 (1146.5, 1400.38)	31.93 (29.04, 35.26)	16.92	446.82 (414.14, 472.55)	21.41 (19.75, 22.62)	930.68 (824.67, 1017.65)	20.17 (17.86, 22.05)	−5.79
**Mortality**										
China	66.24 (52.78, 80.32)	18.56 (15.05, 22.07)	174.40 (133.84, 226.28)	18.95 (14.65, 24.34)	2.10	53.07 (42.22, 64.52)	13.23 (10.70, 16.02)	100.73 (76.60, 128.09)	9.34 (7.10, 11.88)	−29.40
Japan	16.79 (16.13, 17.30)	24.26 (23.20, 25.07)	34.81 (31.70, 36.62)	20.24 (18.75, 21.17)	−16.57	14.56 (13.14, 15.37)	15.27 (13.75, 16.13)	33.11 (24.56, 37.93)	12.17 (9.93, 13.42)	−20.30
South Korea	1.92 (1.59, 2.36)	18.27 (15.41, 22.22)	6.52 (5.21, 7.85)	16.96 (13.38, 20.56)	−7.17	1.89 (1.53, 2.26)	11.91 (9.52, 14.34)	5.14 (3.81, 6.76)	9.37 (7.06, 12.20)	−21.33
North Korea	0.86 (0.61, 1.25)	14.42 (10.66, 20.37)	1.75 (1.19, 2.76)	13.13 (8.94, 20.04)	−8.95	0.92 (0.64, 1.39)	9.96 (7.06, 14.72)	1.66 (1.02, 2.62)	8.63 (5.31, 13.58)	−13.35
Mongolia	0.04 (0.03, 0.05)	8.09 (5.76, 11.25)	0.09 (0.06, 0.12)	9.51 (6.63, 12.67)	17.55	0.04 (0.03, 0.06)	7.43 (5.25, 10.18)	0.09 (0.06, 0.12)	7.11 (4.96, 9.23)	−4.31
United States	33.05 (31.38, 34.20)	25.27 (23.79, 26.23)	39.38 (36.79, 41.23)	15.16 (14.18, 15.88)	−40.01	34.18 (30.33, 36.37)	17.31 (15.60, 18.31)	35.71 (31.05, 38.54)	10.74 (9.54, 11.49)	−37.95
Global	287.71 (269.82, 304.98)	17.72 (16.67, 18.68)	581.56 (528.25, 641.42)	15.35 (13.94, 16.87)	−13.37	282.61 (258.89, 301.42)	13.89 (12.68, 14.80)	462.51 (407.30, 503.54)	9.96 (8.78, 10.84)	−28.29
**DALYs**										
China	2039.86 (1602.51, 2481.12)	454.10 (360.34, 547.99)	4488.27 (3427.06, 5852.47)	452.83 (349.19, 585.28)	−0.28	1525.33 (1200.99, 1884.42)	334.19 (263.37, 410.15)	2360.12 (1798.15, 3027.36)	220.01 (167.51, 282.02)	−34.17
Japan	422.32 (408.29, 435.38)	561.60 (541.80, 579.07)	664.04 (614.92, 698.21)	457.21 (430.95, 478.23)	−18.59	329.87 (306.47, 344.73)	354.64 (330.44, 370.17)	507.55 (405.20, 566.57)	269.77 (236.36, 289.62)	−23.93
South Korea	57.03 (46.65, 71.44)	415.69 (346.92, 512.35)	147.83 (119.92, 178.87)	355.58 (285.90, 430.32)	−14.46	52.01 (42.90, 61.77)	286.41 (234.04, 341.71)	97.08 (74.65, 124.25)	195.26 (152.49, 248.45)	−31.83
North Korea	27.86 (19.50, 41.09)	376.64 (268.85, 547.85)	53.55 (35.61, 86.12)	353.96 (239.17, 555.80)	−6.02	25.89 (17.60, 40.06)	254.49 (174.93, 391.91)	41.55 (25.49, 67.08)	223.55 (137.58, 362.51)	−12.16
Mongolia	1.13 (0.80, 1.57)	210.70 (149.42, 293.20)	2.85 (1.98, 3.86)	243.39 (169.18, 327.91)	15.51	1.25 (0.92, 1.69)	204.36 (146.65, 278.64)	2.55 (1.83, 3.33)	179.09 (126.92, 234.19)	−12.37
United States	765.25 (737.45, 791.07)	571.69 (549.81, 591.11)	942.89 (894.86, 983.33)	378.66 (360.03, 394.79)	−33.76	696.65 (645.88, 732.07)	390.80 (366.98, 408.90)	749.51 (684.02, 794.91)	258.62 (240.28, 272.64)	−33.82
Global	7609.70 (7037.9, 8139.91)	405.58 (378.49, 431.93)	14167.25 (12782.33, 15683.97)	349.67 (316.68, 386.64)	−13.79	6786.95 (6292.86, 7304.11)	316.54 (292.93, 340.38)	10233.85 (9257.56, 11064.62)	224.30 (203.21, 242.65)	−29.14

* ASR, using the GBD standard population.

ASR: Age-standardised rate; DALYs: Disability-adjusted life years; UI: Uncertainty interval.

In 2021, global CRC mortality accounted for 581.56 thousand male deaths and 462.51 thousand female deaths, corresponding to ASMRs of 15.35 per 100,000 in males and 9.96 per 100,000 in females, with both rates showing declines compared with 1990. In East Asia, substantial disparities in mortality burden persisted in 2021, with Japan’s ASMR (the highest) being 2.13 times that of Mongolia (the lowest) for males and 1.71 times for females. Despite these disparities, reductions in ASMRs were observed across most countries. Specifically, rates declined for females in all five countries and for males in all countries except China and Mongolia. Notably, non-overlapping 95% UIs between 1990 and 2021 were found only in Japan for both sexes. Among males, Japan demonstrated the largest percentage decline in ASMR (−16.57%), although its ASMR remained the highest in the region, exceeding that of the United States. In contrast, for females, China exhibited the greatest percentage decline (−29.40%), followed by South Korea, Japan, North Korea and Mongolia. Nevertheless, the percentage decline in ASMRs for both sexes in East Asia was less pronounced than that in the United States (Table 1).

In 2021, global CRC accounted for 14,167.25 thousand DALYs in males and 10,233.85 thousand in females, with ASDRs of 349.67 per 100,000 for males and 224.3 per 100,000 for females, representing reductions of 13.79% (males) and 29.14% (females) since 1990. With the exception of an increase in Mongolian males, ASDRs decreased for both sexes across all other East Asian countries; among these countries, non-overlapping 95% uncertainty intervals between 1990 and 2021 were observed only in Japan for both sexes. Japan experienced the largest percentage reduction in ASDRs among males (−18.59%), while China saw the largest percentage reduction among females (−34.17%), a figure that also exceeded the reductions observed in both the United States and globally. Nevertheless, Japan remained the country with the highest ASDRs in 2021 for both sexes, with its male and female ASDRs being 1.88 and 1.51 times those of Mongolia (the lowest), respectively (Table 1).

### Temporal trends in colorectal cancer burden

**Fig 1:**
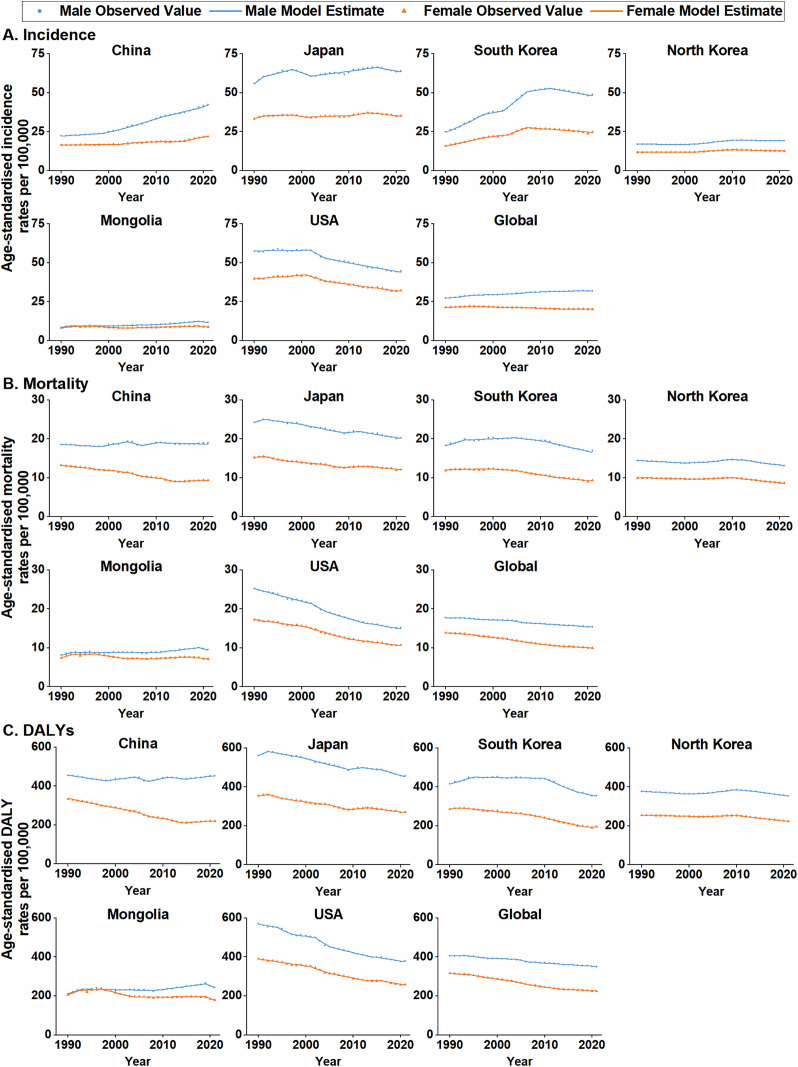
Joinpoint regression analysis of the trends in age-standardised rates of CRC burden in five East Asian countries, the United States, and globally, 1990–2021. (A) Age-standardised incidence rates. (B) Age-standardised mortality rates. (C) Age-standardised DALY rates. Observed values are represented by points for males, triangles for females and solid lines represent model estimates. CRC: colorectal cancer; DALYs: disability-adjusted life years.

Joinpoint regression analysis (Fig 1A, S1 Table) revealed that, based on the AAPCs, statistically significant increases in CRC ASIRs were observed from 1990 to 2021 among males in all East Asian countries and among females in China, South Korea, and North Korea. South Korea experienced the largest annual increases (AAPC: 2.18% in males and 1.41% in females). Notably, male ASIRs followed the global upward trend and female ASIRs diverged from global patterns, with males generally exhibiting higher AAPCs than females. Although increasing trends were observed in female ASIRs in Japan and Mongolia, these changes were not statistically significant. In terms of APC, CRC ASIRs showed distinct phased variations in all countries. In China, male ASIRs steadily increased over the 32-year period, with a significant rise of 3.03% annually from 1999 to 2011 and slowing to 2.04% thereafter. South Korea experienced an initial increase followed by a decline in CRC ASIRs. The fastest growth in South Korean males occurred between 2002 and 2007, followed by a slowdown and a significant annual decline of 1.05% after 2012. Japan, North Korea, and Mongolia showed fluctuating trends, while the most notable changes were observed in Japanese males, with ASIRs markedly rising in the early 1990s, declining from 1998 to 2002, increasing until 2016, and then decreasing again thereafter.

In East Asia, based on AAPCs, CRC ASMRs showed statistically significant declines (1990–2021) among both sexes in Japan, South Korea, and North Korea, and in females in China. Within these populations, females consistently exhibited faster annual reductions than males (e.g., South Korea: −0.88% vs −0.32%). The fastest declines were observed among males in Japan (AAPC = −0.60%) and females in China (AAPC = −1.10%), which were close to the global average but slower than those observed in the United States. Conversely, Mongolian males showed a significant mortality increase (AAPC = 0.50%). Regarding phased changes, ASMR trends in Japan, South Korea and North Korea generally mirrored the corresponding ASIR trends but with earlier onsets and greater magnitudes of decline. Specifically, South Korean male ASMRs increased before 2005 and subsequently decreased, while Japanese ASMRs in males began to decline in the early 1990s. In contrast, the ASMR trends observed in China and Mongolia diverged from their respective ASIR trends. In China, male ASMRs fluctuated between 1990 and 2010 and subsequently declined, while Mongolian male ASMRs initially increased before declining. Remarkably, ASMRs in both sexes in the United States have consistently declined since 1990, with APCs ranging from −3.22% to −1.05%, which is considerably steeper than the reductions observed in East Asian countries (Fig 1B, S2 Table).

Similarly, statistically significant declines in ASDRs were observed for both sexes in Japan, South Korea, and North Korea, and among females in China and Mongolia — a pattern consistent with the global trend and reflected in larger AAPC declines among females. However, Mongolian males exhibited a slight but statistically significant increase (AAPC = 0.44%). Specifically, the ASDRs for Chinese females experienced the largest annual decline (AAPC = −1.35%), approaching the United States level (AAPC = −1.36%) and exceeding the global rate (AAPC = −1.10%). While overall ASDRs decreased in East Asia, phased trends varied across countries, generally mirroring corresponding ASMRs (Fig 1C, S3 Table).

### Age effect on the burden of colorectal cancer

**Fig 2:**
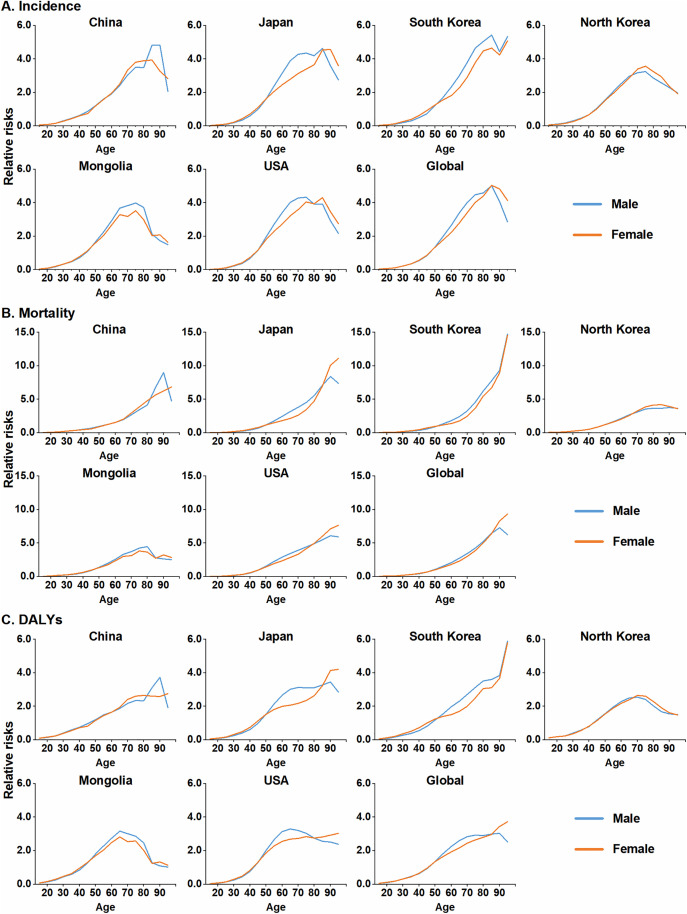
Age-specific relative risks (RRs) for CRC burden by sex in East Asian countries, the United States, and globally, based on the age-period-cohort model. (A) Incidence. (B) Mortality. (C) DALYs. The starting age of the 5-year interval is used to label each age group (e.g., 20 for the 20–24 age group). CRC: colorectal cancer; DALYs: disability-adjusted life years.

After accounting for period and cohort effects, the age-specific RRs of CRC incidence, mortality and DALYs generally increased with age, and distinct regional patterns emerged. Regarding incidence, global CRC RRs increased with age in both sexes, peaking in the 85–89 age group before declining. In East Asia, with the exception of South Korea, CRC trends mirrored the global pattern, although the age at which the peak occurred varied across countries. For instance, in North Korea, male incidence RRs peaked in the 75–79 age group, whereas in Japan, female RRs peaked in the 90–94 age group. South Korea displayed a unique trajectory, with incidence peaking at 85–89 years and a secondary increase in the 95 + age group. Furthermore, CRC incidence RRs were substantially elevated (RRs > 1.0) in individuals aged ≥45 years in Japan, North Korea and Mongolia and in those aged ≥50 years in China and South Korea (Fig 2A, S4 Table).

Regarding CRC mortality and DALYs, global RRs in males peaked in the 90–94 age group before a modest decline, while in females, RRs continued to increase, peaking at 95 + years. In East Asia, CRC mortality and DALY RRs largely mirrored global trends, although sex- and country-specific variations were evident. Specifically, in North Korea and Mongolia, CRC RRs for females peaked and then declined. Remarkably, South Korea was the only country where CRC mortality and DALY RRs demonstrated a continuous increase with age in both sexes, with mortality RRs in the 95 + age group exhibiting a particularly sharp increase (RR 14.72 for males and 14.57 for females) (Fig 2B and 2C, S4 Table).

### Period effect on the burden of colorectal cancer

**Fig 3:**
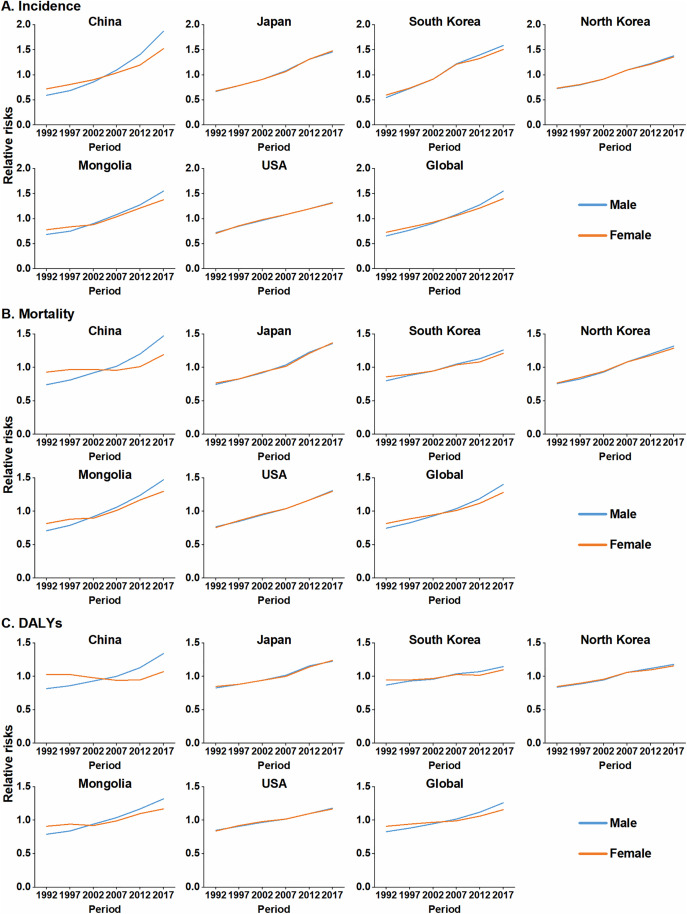
Period-specific relative risks (RRs) for CRC burden by sex in East Asian countries, the United States, and globally, based on the age-period-cohort model. (A) Incidence. (B) Mortality. (C) DALYs. The starting year of the 5-year interval is used to label each period (e.g., 1992 for the 1992–1996 period). CRC: colorectal cancer; DALYs: disability-adjusted life years.

After controlling for age and birth cohort effects, the period-specific RRs of CRC incidence, mortality and DALYs showed an overall increasing trend, peaking in the most recent period (2017–2021) across the studied regions. In East Asia, notable increases in incidence RRs were observed particularly among Chinese males and Korean females (Fig 3A, S5 Table). For mortality and DALYs, pronounced rising trends were observed, for example, among Mongolian males and Japanese females. Notably, China was unique, with female mortality and DALY RRs decreasing between 1997–2001 and 2007–2011. By the final period (2017–2021), the RRs of CRC incidence, mortality and DALYs in China and Mongolia had accelerated considerably (Fig 3B and 3C, S5 Table).

### Cohort effect on the burden of colorectal cancer

**Fig 4:**
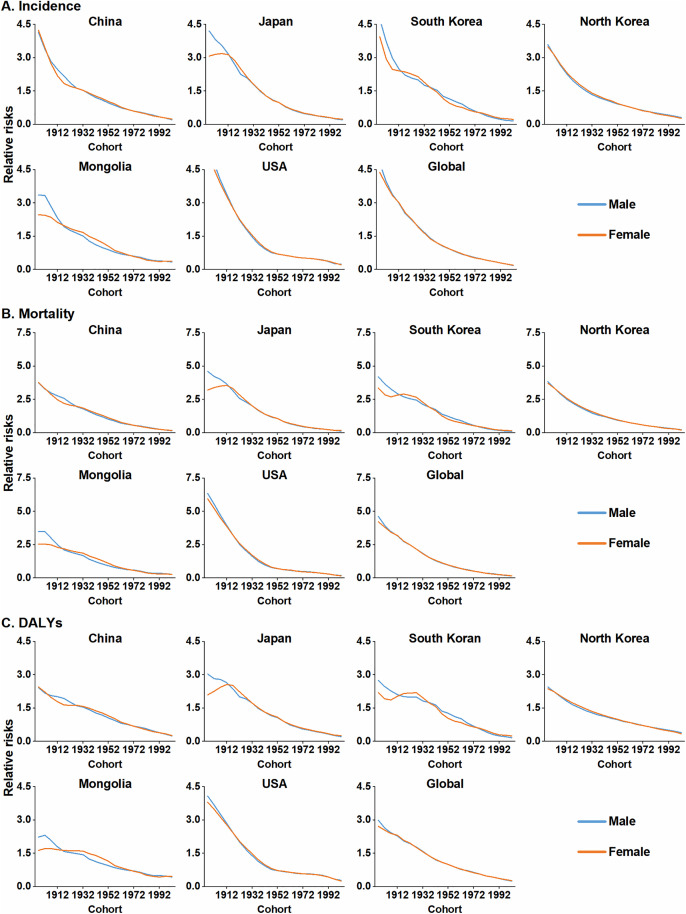
Cohort-specific relative risks (RRs) for CRC burden by sex in East Asian countries, the United States, and globally, based on the age-period-cohort model. (A) Incidence. (B) Mortality. (C) DALYs. The starting year of the 5-year interval is used to label each cohort (e.g., 1912 for the 1912–1916 birth cohort). CRC: colorectal cancer; DALYs: disability-adjusted life years.

After controlling for age and period effects, the cohort-specific RRs of CRC incidence, mortality and DALYs consistently decreased by birth cohort across the studied regions. Globally, CRC RRs initially declined rapidly, with subsequent slower decreases observed across later birth cohorts. The earliest cohort (1897–1901) exhibited the highest CRC RR. Within East Asia, cohort effects generally mirrored the global trend, although notable sex- and country-specific variations were evident. Regarding incidence RRs, Japanese females showed a slight increase in RR from the 1897–1901–1907–1911 cohorts. Minimal changes in incidence RRs were observed in early cohorts of Mongolian males and females (Fig 4A, S6 Table). For mortality and DALY RRs, Japanese and South Korean females experienced an initial increase in earlier cohorts, while Mongolian males and females showed relatively stable mortality RRs and slow increases in DALYs in early cohorts (Fig 4B and 4C, S6 Table).

### Decomposition analysis of colorectal cancer burden

**Fig 5:**
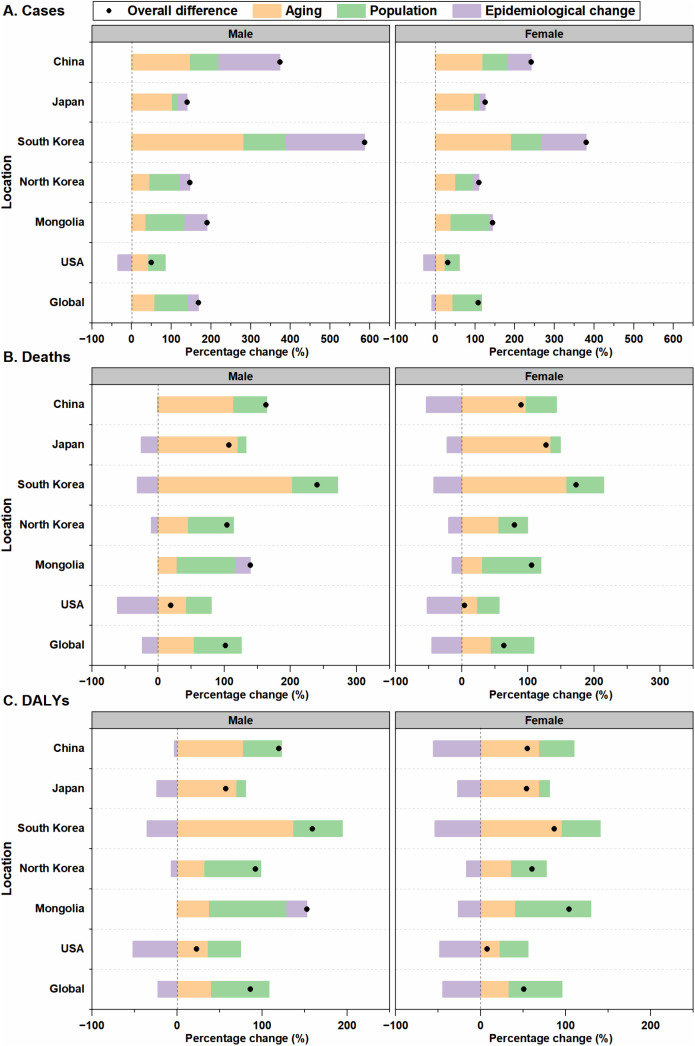
Decomposition of changes in the number of CRC burden in East Asian countries, the United States, and globally, 1990–2021. (A) Cases. (B) Deaths. (C) DALYs. The black dot represents the overall percentage change contributed by all three components — population growth, ageing, and epidemiological change. For each component, the magnitude of a positive value indicates the percentage increase attributed to the component; the magnitude of a negative value indicates the percentage decrease attributed to the related factor. CRC: colorectal cancer; DALYs: disability-adjusted life years.

Fig 5 and S7 Table present the decomposition of changes in the number of new CRC cases, deaths and DALYs from 1990 to 2021. This analysis reveals a notable increase in CRC cases within the five East Asian countries, with South Korea experiencing the largest increases (males: 587.50%; females: 380.26%)—far exceeding those observed in the United States and globally. This was followed by China, Japan, Mongolia, and North Korea. In East Asia, ageing, population growth and epidemiological changes all contributed positively to the increase in CRC cases, with ageing exerting the greatest influence in South Korea (282.43% in males and 191.84% in females). Population growth was the primary driver of the increase in Mongolia (98.68% in males and 97.25% in females), while epidemiological changes had the largest impact on incidence in China (154.22%) (Fig 5A).

Despite a general decline in CRC ASMRs and ASDRs across East Asia from 1990 to 2021, deaths and DALYs still increased, with South Korea experiencing the largest increases. While ageing and population growth contributed to increases in both CRC deaths and DALYs across East Asia, epidemiological changes led to decreases in deaths and DALYs in all groups except Mongolian males, where population growth exerted a greater influence. The most substantial reductions were observed in South Korean males (deaths: −31.35%; DALYs: −35.41%) and Chinese females (deaths: −53.52%; DALYs: −55.41%), both exceeding the global decline levels. (Fig 5B and 5C).

### Association of colorectal cancer burden with SDI

**Fig 6:**
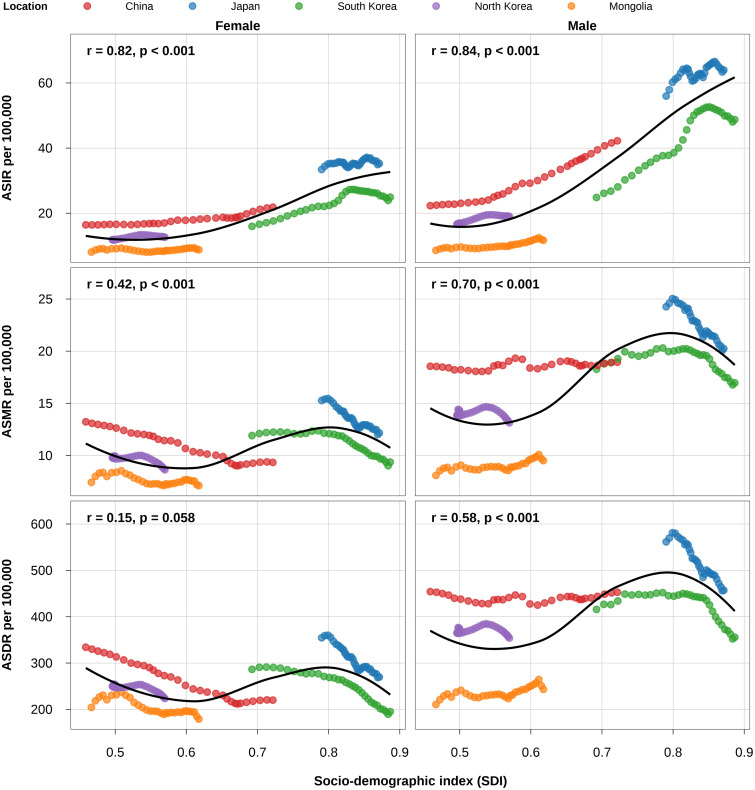
Association between age-standardised CRC burden metrics (ASIR, ASMR, ASDR) and the Socio-Demographic Index (SDI) in five East Asian countries (1990-2021). The solid line shows the Locally Weighted Scatterplot Smoothing (LOESS) regression model fit to all country–year observations. The overall association across all country–year observations is summarised by Spearman’s rank correlation coefficient *r* and the *p*-value. ASIR, age-standardised incidence rate; ASMR, age-standardised mortality rate; ASDR, age-standardised disability-adjusted life year rate.

Across East Asia (1990–2021), CRC burden showed overall positive associations with SDI (Fig 6, S8 Table). Both ASIR and ASMR were positively associated with SDI in both sexes (*r* = 0.42 to 0.84, *p *< 0.001), with stronger associations observed in males. ASDR showed a positive association only in males (*r *= 0.58, *p *< 0.001) and was non-significant in females (*p *= 0.058). LOESS curves revealed a non-linear relationship between CRC burden and SDI, particularly for ASMR and ASDR, which were relatively flat at low-to-middle SDI levels, increased at middle-to-high SDI levels, and then declined at high SDI levels. This pattern was more pronounced in males than in females. Country-specific heterogeneity was marked: ASIR rose with SDI for both sexes in China, Japan, South Korea and North Korea, and for males in Mongolia (*r *= 0.47 to 1.00, *p *< 0.01), with no association in Mongolian females (*p *= 0.215). In contrast, ASMR and ASDR decreased with increasing SDI for both sexes in Japan and South Korea and for females in China, North Korea and Mongolia (*r *= −0.97 to −0.51, *p *< 0.01), whereas Chinese males (ASMR *r *= 0.58) and Mongolian males (ASMR *r *= 0.78; ASDR *r *= 0.73; all *p *< 0.001) showed positive associations with SDI. North Korean males showed no association for ASMR or ASDR, and Chinese males showed no association for ASDR. By contrast, the United States exhibited strong negative associations for all CRC metrics in both sexes (*r *= −1.00 to −0.86, *p *< 0.001).

## Discussion

This study provided an in-depth analysis of CRC burden trends in five East Asian countries from 1990 to 2021 using GBD 2021 data. Joinpoint regression analysis from 1990 to 2021 revealed statistically significant increases in CRC incidence for males in all five East Asian countries and females in China, South Korea and North Korea; statistically significant declines in mortality and DALY rates for both sexes in Japan, South Korea, and North Korea, and for females in China, while Mongolian males showed significant increases; notably, non-overlapping UIs between 1990 and 2021 ASRs were found only for male incidence in China, Japan, and South Korea, and for both-sex mortality and DALY rates in Japan, with overlaps in all other groups. Age-period-cohort analysis revealed a positive association between age and CRC incidence, mortality and DALY RRs, with RRs peaking before declining, except in South Korea, where mortality and DALY RRs continued to increase with advancing age. Period effects generally indicated a linear increase in CRC RRs, except in Chinese females, whose mortality and DALY RRs experienced a brief decline between 1997–2001 and 2007–2011. Analysis of cohort effects revealed a general decline in CRC RRs in more recent birth cohorts across all East Asian countries, although earlier cohorts in Japan and South Korea exhibited elevated RRs. Decomposition analysis demonstrated that ageing and population growth contributed to increases in CRC cases, deaths, and DALYs in East Asian countries, while epidemiological changes contributed to reductions in deaths and DALYs, particularly in China and South Korea. Furthermore, correlation analyses revealed an overall positive association between CRC burden and SDI in East Asia from 1990–2021, with disparities by country and sex.

This study found that the overall CRC incidence rate in East Asia increased from 1990 to 2021, narrowing the incidence gap with the United States, which is consistent with previous findings by Pardamean et al [41]. However, these findings diverge from those based on the GBD 2019 database, which reported elevated CRC mortality in most East Asian countries between 1990 and 2019 [22]. In contrast, the present study, which used data from the updated GBD 2021 database, revealed a decline in mortality over the same period. This discrepancy is likely attributable to substantial methodological refinements and expanded data sources in the 2021 GBD update [42]. These advances integrated more recent and comprehensive epidemiological evidence, improving the accuracy and reliability of mortality estimates. Consequently, our findings—an observed increase in CRC incidence alongside a corresponding decline in mortality and DALYs in most East Asian countries—more accurately reflect the positive developments in CRC screening and treatment as well as the broader impact of socioeconomic development and shifts in lifestyle patterns.

These trends can be explained largely by the phased implementation and maturation of population-based screening programmes, mirroring the experience in Europe where established programmes reduced the CRC burden [43]. The evolution of CRC trends in East Asia between 1990 and 2021 similarly reflects this principle, underscoring the crucial role of organised screening programmes in shaping disease burden. In Japan, the CRC screening programme initiated in 1992 was associated with an initial increase in incidence and a concurrent decrease in mortality, with incidence rates declining after 1998 [44]. Japan has maintained relatively high screening rates: 41.2% among individuals aged ≥40 years in 2019 [45]. Similarly, South Korea integrated CRC screening into its National Cancer Screening Programme (NCSP) in 2004 [46], accelerating incidence while reducing mortality. After 2007, the upward trend in incidence reversed, demonstrating the long-term effectiveness of screening. Screening rates in South Korea reached 64.4% among individuals aged 50–74 years [47]. China implemented government-supported organised cancer screening programmes, initially targeting rural areas in 2005 and subsequently extending to urban areas in 2012 [16]. Although these interventions contributed to later reductions in mortality and DALYs, China’s overall screening rates remain relatively low and the full benefits in reducing incidence are yet to be realised [48]. By comparison, the United States has experienced sustained declines in CRC incidence, mortality and DALY rates, largely attributable to early and widely adopted screening policies introduced in the 1960s [49,50]. Subsequent initiatives, including the National Comprehensive Cancer Control Programme in 1998 and the Colorectal Cancer Control Programme in 2009 [51,52], drove the CRC screening rate among individuals aged ≥50 years from 38% in 2000 to 66% by 2018, thereby contributing to the reduced disease burden [53].

Regarding sex differences, CRC incidence increased more rapidly in males, while mortality and DALYs declined more markedly in females. From a biological perspective, estrogen and progesterone may exert a protective effect by suppressing tumour growth, while testosterone can promote CRC progression [54], partially explaining the higher incidence in males. Behavioural factors also play a major role, as males are more likely to engage in established high-risk behaviours, including smoking and heavy alcohol consumption. Smoking, a well-known CRC risk factor [55], is more prevalent among East Asian males, with rates exceeding 30%, compared with less than 10% in females [56]. Similarly, heavy alcohol consumption primarily increases CRC risk in males [57]. In contrast, females generally exhibit greater health awareness and are more inclined to participate in screening programmes [58], thereby deriving greater benefit from preventive and therapeutic interventions. Consequently, CRC mortality and DALYs declined at a faster rate in females, while the slower rate of improvement observed in males suggests a greater burden of health risks and poorer overall outcomes. This underscores the need for targeted public health strategies for males.

The age effect, as revealed by the age-period-cohort model, demonstrated that CRC incidence, mortality and DALY RRs increase with advancing age, typically peaking and subsequently declining across the evaluated regions. This pattern highlights age as a crucial risk factor for CRC. The decline in immune function associated with ageing increases susceptibility to chronic intestinal diseases and adenomas [59–61], which may progress to CRC over time. However, recent epidemiological trends indicate a rising CRC incidence in younger populations [6], prompting several countries to initiate screening at earlier ages. For instance, Japan’s cancer screening programme recommends CRC screening for individuals aged >40 years [62], while China’s Guideline for the Screening, Early Detection and Early Treatment of Colorectal Cancer recommends screening for individuals aged 50–75 years, and South Korea’s NCSP offers free screening for those aged ≥50 years [63,64]. This shift aligns with the identified high-risk CRC groups in this study: while incidence RRs are highest in older adults, preventive efforts are increasingly targeting younger populations. CRC mortality and DALY RRs peak in older East Asians, particularly in South Korea. According to OECD data, South Korea’s poverty rate among individuals aged >65 years exceeded 40% in 2020 [65], potentially limiting access to prevention and treatment. Meanwhile, the 2021 employment rate for this group was 34.9% [65], suggesting continued financial strain that may delay diagnoses as individuals prioritise income over timely medical care. However, it should be noted that estimates of CRC incidence, mortality, and DALY RRs in older age groups (i.e., 85 years and above) may be affected by limited case numbers and reduced data reliability, particularly given that life expectancy in East Asian countries generally does not exceed 85 years [66]. Therefore, these findings should be interpreted cautiously. Despite rising CRC incidence in younger populations, these findings highlight the continued need for targeted strategies for older adults.

Age-period-cohort model analysis revealed generally increasing CRC incidence, mortality and DALY RRs over time, with downward trends in later birth cohorts. Rising period effects have coincided with rapid economic development and urbanisation, accompanied by dietary shifts towards higher-fat, higher-protein foods and increased consumption of red and processed meat—all established CRC risk factors [7,67]. The accelerated increase in CRC RRs in China and Mongolia may be linked to high red meat consumption [68,69]. In contrast, high-calcium and high-fibre diets can reduce CRC risk [70]. Urbanisation has also increased sedentary behaviour and reduced physical activity, both of which are CRC risk factors [71]. To counter lifestyle-related risks, East Asian countries have strengthened their national preventive programmes in recent years. For instance, China’s 14th Five-Year Plan for National Health Development reinforces the “Three Reductions and Three Healthies” strategy to target CRC dietary risks [72]; Japan’s Health Japan 21 (Third Edition) advocates healthy eating and moderate exercise with quantifiable goals [73]; South Korea’s Healthy Korea 2030 mandates nutrition labelling for processed food and subsidises public fitness infrastructure [74]. From a cohort perspective, governmental health investment has notably increased, particularly in CRC prevention and early screening [75]. For instance, South Korea has expanded screening coverage and promoted widespread FIT testing and colonoscopies [11]. Additionally, advances in liquid biopsy, personalised immunotherapy and robotic surgery have improved diagnosis and treatment [76–78], potentially contributing to better outcomes and reduced cohort-specific RRs.

Decomposition analysis reveals that ageing and population growth as the principal drivers of the increasing CRC burden in East Asia, though epidemiological changes have partially offset their impact on deaths and DALYs. Despite declines in mortality and DALY rates from 1990 to 2021, ageing and population growth still contribute substantially to rising CRC deaths and DALYs, highlighting the need for better health policies and interventions to address demographic shifts. Ageing, population growth and epidemiological changes have all contributed to increased CRC incidence. Ageing has been most prominent in South Korea, while population growth has been more influential in Mongolia, owing to its younger population and higher fertility [15]. As ageing continues, East Asian countries should implement proactive policies to strengthen older adult health management, promote screening and encourage healthy lifestyles [79]. These measures will facilitate earlier detection, reduce disease progression and alleviate the long-term burden of an ageing population.

The association between CRC burden and SDI in East Asia varies by country and sex, reflecting the multidimensional nature of socioeconomic development. Japan and South Korea show a clear “development–mortality decoupling” (where ASMR and ASDR decline as SDI rises)—a pattern consistent with observations in established high‑SDI regions globally [80]. This pattern is likely due to synergistic progress across SDI dimensions: income growth supports healthcare investment [81], improvements in educational attainment enhance health literacy and CRC screening adherence [82], and declining fertility reflects improved women’s empowerment and healthcare access [83]. This synergistic progress has ultimately enabled these nations to translate socioeconomic gains into effective cancer control outcomes. In contrast, China and Mongolia exhibit a male-specific counterintuitive trend: rising CRC mortality and DALYs alongside increasing SDI. This trend potentially reflects imbalanced development: rapid economic growth may be outstripping health education dissemination, particularly in rural areas and among older populations in certain regions [84,85], while traditional gender norms could further contribute to lower health literacy and reduced willingness to seek medical care among men [86]. These contrasting patterns by country and sex suggest that improvements in economic and social indicators alone may be insufficient to achieve widespread reduction and equity in CRC burden across East Asia. Therefore, comprehensive sociodemographic progress must be integrated with equitable, gender-sensitive public health strategies.

Based on the findings, the following strategies are proposed to reduce the CRC burden in the five East Asian countries: First, promote healthy lifestyles by reducing tobacco use, alcohol consumption and other harmful behaviours, while encouraging high-fibre diets and regular physical activity. It is recommended to strengthen regional health promotion efforts in Mongolia to improve dietary habits. Second, expand early screening, particularly for males and the elderly, by appropriately lowering the starting age for screening programmes to improve early detection rates in China, Japan and South Korea. Third, address the challenges of an ageing population by improving healthcare access for the elderly, particularly in South Korea, and implementing targeted health intervention programmes for vulnerable subgroups (e.g., rural populations) in China and Mongolia to ensure equitable health outcomes. Fourth, increase investment in medical innovations, such as AI-assisted colonoscopy and minimally invasive surgery. South Korea and Japan should continue advancing these technologies, while China, North Korea and Mongolia should focus on adoption of these technologies and workforce training. Finally, strengthen CRC data monitoring and analysis, particularly in North Korea and Mongolia, through enhanced international cooperation and capacity building to improve data quality. Furthermore, strategies in China, Japan and South Korea should be adapted to reflect demographic changes and evolving disease burdens.

This study provides valuable insights into East Asian CRC burden through its nuanced exploration of regional variations across five countries. The integration of decomposition and trend analyses provides an innovative approach for quantifying the relative contributions of ageing, population growth and epidemiological changes to observed CRC trends. This methodology identifies primary burden drivers and offers novel insights for effective prevention and control. However, several limitations exist. First, the GBD database relies on modelling estimates, especially in data-scarce areas such as North Korea, where limited local data are supplemented by regional information. This introduces uncertainty, manifesting as wider 95% UIs. Furthermore, because population counts and surveillance data for those aged 85 years and above are often sparse in East Asia, results for individuals in this age group may be less precise and should be interpreted with caution. Second, the joinpoint regression model and age-period-cohort analysis primarily identify trends rather than causal relationships, warranting further research on causal inference. Third, while the decomposition method effectively quantifies the relative contributions of demographic versus non-demographic factors, it cannot disaggregate specific drivers within the epidemiological change contribution. Additionally, although this study quantified the association between SDI and CRC burden, it could not disentangle the relative contributions of SDI components. Finally, limited access to health system data from North Korea and Mongolia constrains a comprehensive assessment of their health systems’ roles in prevention and control. Future studies should investigate the influence of specific, granular socioeconomic factors on CRC burden across East Asia, and examine spatial variations in CRC incidence, mortality and DALYs to enhance understanding.

## Conclusion

In conclusion, joinpoint analysis (1990–2021) revealed statistically significant increases in CRC incidence (males in all five East Asian countries, females in China/South Korea/North Korea) and significant declines in mortality and DALY rates (both sexes in Japan/South Korea/North Korea, Chinese females), but with a rise in Mongolian males; however, substantial changes (non-overlapping UIs) were limited to a few groups, warranting cautious interpretation. The age-period-cohort model revealed that relative risks for CRC incidence, mortality and DALYs generally increased with age and over time periods, while decreasing across successive birth cohorts. Decomposition analysis demonstrated that ageing and population growth were the primary drivers of increased CRC burden. Furthermore, CRC burden was overall positively associated with SDI in East Asia from 1990–2021. Despite some positive trends, the overall burden of CRC remains substantial with distinct age-, sex-, and country-specific patterns. Collectively, these findings underscore the necessity for tailored approaches to CRC control in East Asia, including targeted screening programs, lifestyle interventions specific to males, enhanced elderly healthcare services in rapidly ageing populations such as South Korea, and adaptations to address demographic transitions.

## Supporting information

S1 Table Trends in age-standardised incidence rates of CRC from 1990 to 2021 for males and females in five East Asian countries, the United States, and globally, using the joinpoint regression model. (DOCX)

S2 Table Trends in age-standardised mortality rates of CRC from 1990 to 2021 for males and females in five East Asian countries, the United States, and globally, using the joinpoint regression model. (DOCX)

S3 Table Trends in age-standardised DALY rates of CRC from 1990 to 2021 for males and females in five East Asian countries, the United States, and globally, using the joinpoint regression model. (DOCX)

S4 Table Age-, period-, and cohort-specific relative risks of CRC incidence for males and females in five East Asian countries, the United States, and globally, based on the age-period-cohort model. (DOCX)

S5 Table Age-, period-, and cohort-specific relative risks of CRC mortality for males and females in five East Asian countries, the United States, and globally, based on the age-period-cohort model. (DOCX)

S6 Table Age-, period-, and cohort-specific relative risks of CRC DALYs for males and females in five East Asian countries, the United States, and globally, based on the age-period-cohort model. (DOCX)

S7 Table Changes in CRC cases, deaths, and DALY numbers according to population-level determinants of ageing, population growth, and epidemiological changes from 1990 to 2021 in five East Asian countries, the United States, and globally for males and females. (DOCX)

S8 Table Spearman’s rank correlation coefficient between age-standardised CRC burden and SDI across five East Asian countries, the United States, and globally from 1990 to 2021. (DOCX)

## Abbreviations

ASDR: age-standardised DALY rate
ASIR: age-standardised incidence rate
ASMR: age-standardised mortality rate
ASR: age-standardised rate
APC: annual percent change
AAPC: average annual percent change
CRC: colorectal cancer
CI: confidence interval
DALY: disability-adjusted life year
GBD: Global Burden of Disease
NCCR: National Central Cancer Registry
RR: relative risk
UI: uncertainty intervals
