# Supplementary material for: Trends in incidence, mortality and disability-adjusted life years of colorectal cancer in East Asia (1990–2021): An analysis of the Global Burden of Disease study 2021
Source: PLoS One. 2025 Oct 8;20(10):e0334229. doi: 10.1371/journal.pone.0334229 (PMC12507298; doi:10.1371/journal.pone.0334229)
Supplement: S1 Table — (DOCX) [file pone.0334229.s001.docx]

**S1 Table.** **Trends in age-****standardised incidence rates of CRC from 1990 to 2021 for males and females in five East Asian countries, the United States, and globally, using the joinpoint regression model**

| **Countries** | **Trend 1** | | **Trend 2** | | **Trend 3** | | **Trend 4** | | | | **Trend 5** | | | | **Trend 6** | | **1990–2021** |
| --- | --- | --- | --- | --- | --- | --- | --- | --- | --- | --- | --- | --- | --- | --- | --- | --- | --- |
|  | **Period** | **APC (95% CI), %** | **Period** | **APC (95% CI), %** | **Period** | **APC (95% CI), %** | **Period** | | **APC (95% CI), %** | | **Period** | | **APC (95% CI), %** | | **Period** | **APC (95% CI), %** | **AAPC (95% CI), %** |
| **Male** | | | | | | | | | | | | | | | | | |
| China | 1990−1999 | 0.88^*^ (0.59, 1.17) | 1999−2011 | 3.03^*^ (2.87, 3.19) | 2011−2021 | 2.04^*^ (1.74, 2.34) | |  | |  | |  | |  |  |  | 2.08^*^ (1.95, 2.22) |
| Japan | 1990–1992 | 3.92^*^ (1.49, 6.40) | 1992–1998 | 1.27^*^ (0.71, 1.84) | 1998–2002 | −1.75^*^ (−3.03, −0.46) | | 2002–2016 | | 0.64^*^ (0.48, 0.81) | | 2016–2021 | | −0.92^*^ (−1.76, −0.07) |  |  | 0.40^*^ (0.13, 0.68) |
| South Korea | 1990–1998 | 4.90^*^ (4.62, 5.18) | 1998–2002 | 1.45^*^ (0.38, 2.53) | 2002–2007 | 5.69^*^ (5.04, 6.35) | | 2007–2012 | | 0.89^*^ (0.26, 1.54) | | 2012–2021 | | −1.05^*^ (−1.27, −0.82) |  |  | 2.18^*^ (1.97, 2.38) |
| North Korea | 1990–1993 | 0.13 (−0.15, 0.41) | 1993–1999 | −0.29^*^ (−0.42, −0.17) | 1999–2002 | 0.63^*^ (0.01, 1.25) | | 2002–2010 | | 1.82^*^ (1.74, 1.91) | | 2010–2021 | | −0.27^*^ (−0.32, −0.23) |  |  | 0.39^*^ (0.32, 0.46) |
| Mongolia | 1990–1992 | 4.55^*^ (0.40, 8.87) | 1992–2000 | −0.21 (−0.74, 0.32) | 2000–2011 | 1.02^*^ (0.67, 1.36) | | 2011–2019 | | 2.26^*^ (1.74, 2.79) | | 2019–2021 | | −2.96 (−6.65, 0.89) |  |  | 0.98^*^ (0.58, 1.38) |
| United States | 1990–2002 | 0.06 (−0.09, 0.22) | 2002–2005 | −3.01^*^ (−5.53, −0.42) | 2005–2021 | −1.15^*^ (−1.25, −1.05) | |  | |  | |  | |  |  |  | −0.86^*^ (−1.12, −0.61) |
| Global | 1990–1996 | 1.10^*^ (0.99, 1.21) | 1996–2002 | 0.30^*^ (0.18, 0.43) | 2002–2010 | 0.69^*^ (0.61, 0.78) | | 2010–2021 | | 0.19^*^ (0.12, 0.26) | |  | |  |  |  | 0.52^*^ (0.47, 0.56) |
| **Female** | | | | | | | | | | | | | | | | | |
| China | 1990–2002 | 0.21^*^ (0.11, 0.31) | 2002–2005 | 1.87^*^ (0.55, 3.21) | 2005–2011 | 0.78^*^ (0.50, 1.07) | | 2011–2015 | | 0.06 (−0.72, 0.85) | | 2015–2021 | | 2.93^*^ (2.54, 3.33) |  |  | 0.98^*^ (0.81, 1.16) |
| Japan | 1990–1992 | 2.47^*^ (0.38, 4.59) | 1992–1998 | 0.31 (−0.19, 0.81) | 1998–2001 | −1.33 (−3.82, 1.22) | | 2001–2010 | | 0.26 (−0.03, 0.56) | | 2010–2014 | | 1.46 (−0.08, 3.02) | 2014–2021 | −0.87^*^ (−1.34, −0.40) | 0.15 (−0.20, 0.51) |
| South Korea | 1990–1998 | 3.66^*^ (3.34, 3.97) | 1998–2003 | 1.54^*^ (0.73, 2.36) | 2003–2007 | 4.81^*^ (3.53, 6.10) | | 2007–2021 | | −0.83^*^ (−0.98, −0.68) | |  | |  |  |  | 1.41^*^ (1.20, 1.63) |
| North Korea | 1990–1994 | 0.31^*^ (0.16, 0.45) | 1994–1999 | −0.09 (−0.24, 0.05) | 1999–2002 | 0.27 (−0.22, 0.75) | | 2002–2010 | | 1.54^*^ (1.47, 1.61) | | 2010–2015 | | −0.80^*^ (−0.96, −0.64) | 2015–2021 | −0.42^*^ (−0.51, −0.33) | 0.23^*^ (0.17, 0.29) |
| Mongolia | 1990–1992 | 4.97^*^ (1.13, 8.95) | 1992–1997 | 0.48 (−0.60, 1.57) | 1997–2003 | −2.25^*^ (−3.08, −1.41) | | 2003–2019 | | 1.00^*^ (0.85, 1.16) | | 2019–2021 | | −3.68^*^ (−6.90, −0.35) |  |  | 0.22 (−0.16, 0.61) |
| United States | 1990–2001 | 0.52^*^ (0.36, 0.69) | 2001–2005 | −2.35^*^ (−3.58, −1.11) | 2005–2021 | −1.18^*^ (−1.28, −1.08) | |  | |  | |  | |  |  |  | −0.73^*^ (−0.90, −0.56) |
| Global | 1990–1995 | 0.61^*^ (0.45, 0.78) | 1995–1999 | −0.30 (−0.65, 0.06) | 1999–2002 | −0.68 (−1.39, 0.03) | | 2002–2008 | | −0.18^*^ (−0.35, −0.02) | | 2008–2013 | | −0.69^*^ (−0.94, −0.44) | 2013–2021 | −0.11 (−0.22, 0.00) | −0.18^*^ (−0.28, −0.08) |

^*^ Indicates that the APC or AAPC is significantly different from zero at the alpha = 0.05 level.

AAPC: average annual percentage change; APC: annual percentage change; CI: confidence interval.
