# Supplementary material for: Trends in incidence, mortality and disability-adjusted life years of colorectal cancer in East Asia (1990–2021): An analysis of the Global Burden of Disease study 2021
Source: PLoS One. 2025 Oct 8;20(10):e0334229. doi: 10.1371/journal.pone.0334229 (PMC12507298; doi:10.1371/journal.pone.0334229)
Supplement: S2 Table — (DOCX) [file pone.0334229.s002.docx]

**S2 Table.** **Trends in age-standardised mortality rates of CRC** **from 1990 to 2021 for males and females in five East Asian countries, the United States, and globally, using the joinpoint regression model**

| **Countries** | **Trend 1** | | | **Trend 2** | | | | **Trend 3** | | | | **Trend 4** | | | | **Trend 5** | | | | **Trend 6** | | | | **1990–2021** | |
| --- | --- | --- | --- | --- | --- | --- | --- | --- | --- | --- | --- | --- | --- | --- | --- | --- | --- | --- | --- | --- | --- | --- | --- | --- | --- |
|  | **Period** | **APC (95% CI), %** | | **Period** | | **APC (95% CI), %** | | **Period** | | **APC (95% CI), %** | | **Period** | | **APC (95% CI), %** | | **Period** | | **APC (95% CI), %** | | **Period** | | **APC (95% CI), %** | | **AAPC (95% CI), %** | |
| **Male** | | | | | | | | | | | | | | | | | | | | | | | | | |
| China | 1990–1998 | | −0.40^*^ (−0.66, −0.15) | | 1998–2004 | | 1.19^*^ (0.81, 1.56) | | 2004–2007 | | −1.85^*^ (−3.37, −0.30) | | 2007–2010 | | 1.25 (−0.45, 2.98) | | 2010–2021 | | −0.16 (−0.34, 0.03) | |  | |  | | 0.01 (−0.23, 0.24) |
| Japan | 1990–1992 | | 1.55^*^ (0.00, 3.12) | | 1992–1999 | | −0.63^*^ (−0.91, −0.36) | | 1999–2009 | | −1.05^*^ (−1.23, −0.87) | | 2009–2012 | | 0.63 (−1.41, 2.72) | | 2012–2021 | | −0.95^*^ (−1.15, −0.75) | |  | |  | | −0.60^*^ (−0.82, −0.37) |
| South Korea | 1990–1994 | | 1.82^*^ (0.94, 2.71) | | 1994–2005 | | 0.26^*^ (0.07, 0.44) | | 2005–2011 | | −0.73^*^ (−1.21, −0.25) | | 2011–2021 | | −1.55^*^ (−1.78, −1.33) | |  | |  | |  | |  | | −0.32^*^ (−0.49, −0.16) |
| North Korea | 1990–2000 | | −0.49^*^ (−0.52, −0.45) | | 2000–2004 | | 0.39^*^ (0.16, 0.63) | | 2004–2010 | | 0.86^*^ (0.76, 0.97) | | 2010–2013 | | −0.56^*^ (−1.03, −0.10) | | 2013–2018 | | −1.32^*^ (−1.47, −1.18) | | 2018–2021 | | −0.96^*^ (−1.23, −0.70) | | −0.30^*^ (−0.37, −0.24) |
| Mongolia | 1990–1992 | | 4.02 (−0.62, 8.89) | | 1992–2010 | | 0.00 (−0.16, 0.16) | | 2010–2019 | | 1.50^*^ (1.02, 1.99) | | 2019–2021 | | −2.93 (−6.96, 1.28) | |  | |  | |  | |  | | 0.50^*^ (0.08, 0.91) |
| United States | 1990–2002 | | −1.38^*^ (−1.52, −1.23) | | 2002–2005 | | −3.22^*^ (−5.72, −0.65) | | 2005–2013 | | −2.00^*^ (−2.34, −1.65) | | 2013–2021 | | −1.23^*^ (−1.54, −0.93) | |  | |  | |  | |  | | −1.68^*^ (−1.95, −1.41) |
| Global | 1990–1994 | | −0.06 (−0.28, 0.17) | | 1994–1997 | | −0.73^*^ (−1.35, −0.11) | | 1997–2004 | | −0.23^*^ (−0.33, −0.14) | | 2004–2007 | | −1.30^*^ (−1.84, −0.77) | | 2007–2010 | | −0.23 (−0.84, 0.39) | | 2010–2021 | | −0.51^*^ (−0.57, −0.45) | | −0.46^*^ (−0.56, −0.36) |
| **Female** | | | | | | | | | | | | | | | | | | | | | | | | | |
| China | 1990–2004 | | −1.07^*^ (−1.14, −1.00) | | 2004–2007 | | −3.01^*^ (−4.04, −1.97) | | 2007–2011 | | −1.29^*^ (−1.91, −0.67) | | 2011–2014 | | −3.07^*^ (−4.55, −1.57) | | 2014–2021 | | 0.66^*^ (0.38, 0.95) | |  | |  | | −1.10^*^ (−1.29, −0.90) |
| Japan | 1990–1992 | | 0.76 (−0.79, 2.33) | | 1992–1996 | | −1.71^*^ (−2.53, −0.88) | | 1996–2005 | | −0.87^*^ (−1.09, −0.64) | | 2005–2008 | | −1.90 (−4.13, 0.39) | | 2008–2014 | | 0.45 (−0.10, 1.01) | | 2014–2021 | | −1.01^*^ (−1.36, −0.65) | | −0.75^*^ (−1.03, −0.47) |
| South Korea | 1990–2000 | | 0.17 (−0.03, 0.37) | | 2000–2005 | | −0.74 (−1.52, 0.04) | | 2005–2013 | | −1.81^*^ (−2.13, −1.49) | | 2013–2021 | | −1.35^*^ (−1.72, −0.98) | |  | |  | |  | |  | | −0.88^*^ (−1.06, −0.70) |
| North Korea | 1990–1995 | | −0.13^*^ (−0.23, −0.04) | | 1995–2002 | | −0.32^*^ (−0.40, −0.25) | | 2002–2005 | | 0.33 (−0.13, 0.80) | | 2005–2010 | | 0.61^*^ (0.46, 0.75) | | 2010–2021 | | −1.40^*^ (−1.44, −1.37) | |  | |  | | −0.47^*^ (−0.52, −0.41) |
| Mongolia | 1990–1992 | | 4.85^*^ (0.52, 9.37) | | 1992–1997 | | 0.49 (−0.70, 1.71) | | 1997–2003 | | −2.31^*^ (−3.23, −1.38) | | 2003–2008 | | −0.52 (−1.87, 0.84) | | 2008–2017 | | 0.85^*^ (0.42, 1.27) | | 2017–2021 | | −1.93^*^ (−3.07, −0.78) | | −0.16 (−0.60, 0.28) |
| United States | 1990–2001 | | −1.05^*^ (−1.22, −0.89) | | 2001–2010 | | −2.46^*^ (−2.74, −2.19) | | 2010–2021 | | −1.35^*^ (−1.53, −1.17) | |  | |  | |  | |  | |  | |  | | −1.57^*^ (−1.68, −1.46) |
| Global | 1990–1994 | | −0.51^*^ (−0.79, −0.23) | | 1994–2003 | | −1.11^*^ (−1.20, −1.03) | | 2003–2007 | | −1.76^*^ (−2.18, −1.34) | | 2007–2013 | | −1.35^*^ (−1.55, −1.15) | | 2013–2021 | | −0.69^*^ (−0.81, −0.57) | |  | |  | | −1.06^*^ (−1.14, −0.98) |

^*^ Indicates that the APC or AAPC is significantly different from zero at the alpha = 0.05 level.

AAPC: average annual percentage change; APC: annual percentage change; CI: confidence interval.
