# Supplementary material for: Trends in incidence, mortality and disability-adjusted life years of colorectal cancer in East Asia (1990–2021): An analysis of the Global Burden of Disease study 2021
Source: PLoS One. 2025 Oct 8;20(10):e0334229. doi: 10.1371/journal.pone.0334229 (PMC12507298; doi:10.1371/journal.pone.0334229)
Supplement: S3 Table — (DOCX) [file pone.0334229.s003.docx]

**S3 Table.** **Trends in age-standardised DALY rates** **of CRC from 1990 to 2021 for males and females in five East Asian countries, the United States, and globally, using the joinpoint regression model**

|  | **Trend 1** | | **Trend 2** | | **Trend 3** | | **Trend 4** | | **Trend 5** | | **Trend 6** | | **1990–2021** |
| --- | --- | --- | --- | --- | --- | --- | --- | --- | --- | --- | --- | --- | --- |
| **Countries** | **Period** | **APC (95% CI), %** | **Period** | **APC (95% CI), %** | **Period** | **APC (95% CI), %** | **Period** | **APC (95% CI), %** | **Period** | **APC (95% CI), %** | **Period** | **APC (95% CI), %** | **AAPC (95% CI), %** |
| **Male** | | | | | | | | | | | | | |
| China | 1990–1998 | −0.82^*^ (−0.96, −0.67) | 1998–2004 | 0.73^*^ (0.53, 0.93) | 2004–2007 | −1.65^*^ (−2.50, −0.80) | 2007–2011 | 1.21^*^ (0.73, 1.69) | 2011–2015 | −0.52 (−1.10, 0.05) | 2015–2021 | 0.66^*^ (0.36, 0.95) | −0.02 (−0.16, 0.12) |
| Japan | 1990–1992 | 1.84^*^ (0.45, 3.25) | 1992–1999 | −0.76^*^ (−0.99, −0.52) | 1999–2009 | −1.18^*^ (−1.33, −1.03) | 2009–2012 | 0.63 (−1.13, 2.41) | 2012–2016 | −0.62 (−1.53, 0.30) | 2016–2021 | −1.44^*^ (−1.89, −0.99) | −0.69^*^ (−0.91, −0.46) |
| South Korea | 1990–1995 | 1.55^*^ (1.02, 2.08) | 1995–2010 | −0.09^*^ (−0.18, 0.00) | 2010–2017 | −2.46^*^ (−2.80, −2.13) | 2017–2021 | −1.39^*^ (−2.16, −0.62) |  |  |  |  | −0.54^*^ (−0.69, −0.39) |
| North Korea | 1990–2000 | −0.37^*^ (−0.40, −0.34) | 2000–2004 | 0.31^*^ (0.13, 0.50) | 2004–2010 | 0.79^*^ (0.70, 0.88) | 2010–2013 | −0.59^*^ (−0.96, −0.22) | 2013–2021 | −0.86^*^ (−0.90, −0.82) |  |  | −0.21^*^ (−0.25, −0.16) |
| Mongolia | 1990–1993 | 3.33^*^ (1.11, 5.60) | 1993–2008 | −0.21^*^ (−0.42, 0.00) | 2008–2019 | 1.29^*^ (0.95, 1.63) | 2019–2021 | −3.56 (−7.42, 0.46) |  |  |  |  | 0.44^*^ (0.08, 0.79) |
| United States | 1990–1994 | −0.73^*^ (−1.21, −0.24) | 1994–1997 | −2.18^*^ (−3.77, −0.58) | 1997–2002 | −0.67^*^ (−1.20, −0.14) | 2002–2005 | −3.28^*^ (−4.90, −1.64) | 2005–2013 | −1.35^*^ (−1.57, −1.13) | 2013–2021 | −0.92^*^ (−1.12, −0.73) | −1.32^*^ (−1.56, −1.08) |
| Global | 1990–1994 | 0.05 (−0.22, 0.32) | 1994–1997 | −0.90^*^ (−1.57, −0.22) | 1997–2004 | −0.31^*^ (−0.41, −0.20) | 2004–2007 | −1.16^*^ (−1.72, −0.58) | 2007–2021 | −0.45^*^ (−0.50, −0.41) |  |  | −0.47^*^ (−0.56, −0.38) |
| **Female** | | | | | | | | | | | | | |
| China | 1990–2004 | −1.55^*^ (−1.63, −1.48) | 2004–2007 | −3.38^*^ (−4.42, −2.32) | 2007–2011 | −1.47^*^ (−2.12, −0.82) | 2011–2014 | −2.81^*^ (−4.32, −1.27) | 2014–2021 | 0.65^*^ (0.35, 0.96) |  |  | −1.35^*^ (−1.55, −1.15) |
| Japan | 1990–1992 | 0.86 (−0.44, 2.16) | 1992–1995 | −1.91^*^ (−3.23, −0.58) | 1995–2005 | −1.05^*^ (−1.19, −0.91) | 2005–2009 | −2.01^*^ (−2.89, −1.12) | 2009–2013 | 1.00^*^ (0.03, 1.97) | 2013–2021 | −1.12^*^ (−1.35, −0.89) | −0.89^*^ (−1.11, −0.67) |
| South Korea | 1990–1992 | 0.95 (−0.94, 2.86) | 1992–2005 | −0.82^*^ (−0.91, −0.72) | 2005–2009 | −1.57^*^ (−2.27, −0.86) | 2009–2019 | −2.44^*^ (−2.60, −2.27) | 2019–2021 | 0.10 (−2.32, 2.58) |  |  | −1.27^*^ (−1.48, −1.06) |
| North Korea | 1990–1995 | −0.15^*^ (−0.21, −0.09) | 1995–2003 | −0.32^*^ (−0.36, −0.29) | 2003–2010 | 0.46^*^ (0.41, 0.50) | 2010–2017 | −1.21^*^ (−1.25, −1.16) | 2017–2021 | −1.05^*^ (−1.15, −0.96) |  |  | −0.41^*^ (−0.44, −0.39) |
| Mongolia | 1990–1992 | 4.56^*^ (0.42, 8.88) | 1992–1997 | 0.88 (−0.26, 2.03) | 1997–2004 | −2.75^*^ (−3.42, −2.08) | 2004–2019 | 0.05 (−0.13, 0.22) | 2019–2021 | −4.32^*^ (−7.66, −0.85) |  |  | −0.46^*^ (−0.86, −0.06) |
| United States | 1990–2001 | −0.97^*^ (−1.13, −0.80) | 2001–2004 | −2.74^*^ (−5.20, −0.22) | 2004–2013 | −1.66^*^ (−1.94, −1.38) | 2013–2016 | −0.05 (−2.65, 2.62) | 2016–2021 | −1.62^*^ (−2.20, −1.04) |  |  | −1.36^*^ (−1.71, −1.01) |
| Global | 1990–1994 | −0.61^*^ (−0.94, −0.27) | 1994–2003 | −1.23^*^ (−1.32, −1.14) | 2003–2007 | −1.88^*^ (−2.30, −1.45) | 2007–2013 | −1.38^*^ (−1.57, −1.19) | 2013–2021 | −0.60^*^ (−0.72, −0.47) |  |  | −1.10^*^ (−1.18, −1.02) |

^*^ Indicates that the APC or AAPC is significantly different from zero at the alpha = 0.05 level.

AAPC: average annual percentage change; APC: annual percentage change; CI: confidence interval; DALY: disability-adjusted life year.
