# Supplementary material for: Trends in incidence, mortality and disability-adjusted life years of colorectal cancer in East Asia (1990–2021): An analysis of the Global Burden of Disease study 2021
Source: PLoS One. 2025 Oct 8;20(10):e0334229. doi: 10.1371/journal.pone.0334229 (PMC12507298; doi:10.1371/journal.pone.0334229)
Supplement: S4 Table — (DOCX) [file pone.0334229.s004.docx]

**S4 Table.** **Age-, period-, and cohort-specific relative risks of CRC incidence for males and females in five East Asian countries, the United States, and globally, based on the age-period-cohort model**

|  | **China** | | **Japan** | | **South Korea** | | **North Korea** | | **Mongolia** | | **United States** | | **Global** | |
| --- | --- | --- | --- | --- | --- | --- | --- | --- | --- | --- | --- | --- | --- | --- |
|  | **Male** | **Female** | **Male** | **Female** | **Male** | **Female** | **Male** | **Female** | **Male** | **Female** | **Male** | **Female** | **Male** | **Female** |
|  | **RR (95% CI)** | **RR (95% CI)** | **RR (95% CI)** | **RR (95% CI)** | **RR (95% CI)** | **RR (95% CI)** | **RR (95% CI)** | **RR (95% CI)** | **RR (95% CI)** | **RR (95% CI)** | **RR (95% CI)** | **RR (95% CI)** | **RR (95% CI)** | **RR (95% CI)** |
| **Age** | | | | | | | | | | | | | | |
| 15-19 | 0.06 (0.05, 0.06) | 0.06 (0.06, 0.07) | 0.03 (0.03, 0.03) | 0.03 (0.03, 0.03) | 0.04 (0.03, 0.04) | 0.04 (0.03, 0.04) | 0.07 (0.07, 0.07) | 0.07 (0.07, 0.07) | 0.05 (0.04, 0.05) | 0.05 (0.05, 0.05) | 0.02 (0.02, 0.02) | 0.02 (0.02, 0.02) | 0.04 (0.03, 0.04) | 0.04 (0.04, 0.04) |
| 20-24 | 0.09 (0.08, 0.10) | 0.10 (0.09, 0.11) | 0.05 (0.05, 0.05) | 0.06 (0.05, 0.06) | 0.06 (0.05, 0.06) | 0.07 (0.06, 0.07) | 0.11 (0.10, 0.11) | 0.10 (0.10, 0.11) | 0.08 (0.08, 0.09) | 0.09 (0.09, 0.10) | 0.05 (0.05, 0.05) | 0.05 (0.05, 0.05) | 0.06 (0.06, 0.06) | 0.07 (0.07, 0.07) |
| 25-29 | 0.16 (0.15, 0.17) | 0.16 (0.15, 0.17) | 0.09 (0.09, 0.10) | 0.11 (0.11, 0.12) | 0.12 (0.11, 0.13) | 0.14 (0.13, 0.15) | 0.17 (0.16, 0.17) | 0.15 (0.15, 0.16) | 0.17 (0.16, 0.18) | 0.20 (0.19, 0.21) | 0.10 (0.10, 0.11) | 0.12 (0.11, 0.12) | 0.11 (0.11, 0.12) | 0.12 (0.12, 0.12) |
| 30-34 | 0.30 (0.28, 0.33) | 0.29 (0.27, 0.31) | 0.20 (0.19, 0.20) | 0.23 (0.22, 0.24) | 0.21 (0.19, 0.23) | 0.27 (0.25, 0.29) | 0.30 (0.29, 0.30) | 0.27 (0.26, 0.28) | 0.33 (0.30, 0.35) | 0.33 (0.32, 0.35) | 0.22 (0.21, 0.22) | 0.24 (0.24, 0.25) | 0.22 (0.21, 0.23) | 0.22 (0.22, 0.23) |
| 35-39 | 0.45 (0.42, 0.49) | 0.43 (0.41, 0.46) | 0.36 (0.35, 0.37) | 0.44 (0.42, 0.46) | 0.31 (0.28, 0.34) | 0.40 (0.37, 0.44) | 0.45 (0.44, 0.47) | 0.43 (0.42, 0.45) | 0.48 (0.45, 0.51) | 0.51 (0.48, 0.54) | 0.37 (0.36, 0.38) | 0.41 (0.40, 0.42) | 0.35 (0.34, 0.36) | 0.35 (0.34, 0.36) |
| 40-44 | 0.64 (0.59, 0.70) | 0.61 (0.58, 0.66) | 0.62 (0.60, 0.64) | 0.71 (0.68, 0.74) | 0.50 (0.45, 0.55) | 0.64 (0.59, 0.69) | 0.67 (0.65, 0.69) | 0.68 (0.66, 0.70) | 0.71 (0.67, 0.76) | 0.79 (0.75, 0.84) | 0.68 (0.66, 0.70) | 0.73 (0.71, 0.75) | 0.54 (0.52, 0.56) | 0.56 (0.55, 0.57) |
| 45-49 | 0.86 (0.79, 0.93) | 0.75 (0.70, 0.80) | 1.00 (0.96, 1.03) | 1.10 (1.06, 1.16) | 0.75 (0.69, 0.83) | 0.94 (0.87, 1.02) | 1.09 (1.05, 1.12) | 1.04 (1.00, 1.07) | 1.09 (1.02, 1.17) | 1.13 (1.07, 1.20) | 1.17 (1.13, 1.20) | 1.18 (1.15, 1.22) | 0.85 (0.82, 0.88) | 0.86 (0.84, 0.87) |
| 50-54 | 1.21 (1.11, 1.31) | 1.19 (1.12, 1.28) | 1.61 (1.55, 1.67) | 1.63 (1.55, 1.70) | 1.21 (1.10, 1.34) | 1.27 (1.17, 1.38) | 1.56 (1.51, 1.61) | 1.53 (1.48, 1.58) | 1.67 (1.56, 1.79) | 1.61 (1.52, 1.70) | 1.96 (1.91, 2.02) | 1.80 (1.75, 1.86) | 1.37 (1.33, 1.42) | 1.34 (1.31, 1.37) |
| 55-59 | 1.60 (1.48, 1.74) | 1.59 (1.49, 1.70) | 2.39 (2.30, 2.48) | 2.05 (1.96, 2.14) | 1.72 (1.56, 1.89) | 1.56 (1.44, 1.70) | 2.07 (2.00, 2.14) | 1.96 (1.90, 2.03) | 2.26 (2.11, 2.42) | 2.04 (1.92, 2.16) | 2.71 (2.63, 2.80) | 2.29 (2.23, 2.36) | 1.99 (1.92, 2.05) | 1.78 (1.75, 1.82) |
| 60-64 | 1.94 (1.78, 2.11) | 1.97 (1.84, 2.10) | 3.17 (3.06, 3.30) | 2.47 (2.36, 2.59) | 2.34 (2.12, 2.57) | 1.83 (1.68, 1.99) | 2.54 (2.46, 2.62) | 2.41 (2.33, 2.49) | 2.94 (2.74, 3.15) | 2.69 (2.54, 2.85) | 3.42 (3.32, 3.53) | 2.74 (2.67, 2.82) | 2.66 (2.57, 2.75) | 2.23 (2.19, 2.28) |
| 65-69 | 2.42 (2.23, 2.63) | 2.51 (2.35, 2.68) | 3.89 (3.74, 4.03) | 2.79 (2.67, 2.92) | 3.02 (2.74, 3.32) | 2.28 (2.10, 2.47) | 2.97 (2.88, 3.07) | 2.87 (2.79, 2.96) | 3.68 (3.44, 3.94) | 3.30 (3.11, 3.49) | 4.02 (3.90, 4.14) | 3.23 (3.14, 3.33) | 3.39 (3.28, 3.50) | 2.79 (2.74, 2.85) |
| 70-74 | 3.06 (2.82, 3.31) | 3.35 (3.14, 3.57) | 4.29 (4.14, 4.45) | 3.14 (3.01, 3.29) | 3.82 (3.48, 4.19) | 2.92 (2.69, 3.17) | 3.18 (3.08, 3.29) | 3.41 (3.31, 3.52) | 3.83 (3.59, 4.10) | 3.18 (3.01, 3.36) | 4.29 (4.17, 4.41) | 3.60 (3.50, 3.70) | 4.03 (3.90, 4.16) | 3.42 (3.35, 3.48) |
| 75-79 | 3.50 (3.23, 3.78) | 3.82 (3.59, 4.07) | 4.36 (4.21, 4.52) | 3.40 (3.26, 3.56) | 4.65 (4.24, 5.09) | 3.80 (3.51, 4.11) | 3.25 (3.15, 3.35) | 3.57 (3.46, 3.68) | 3.98 (3.72, 4.24) | 3.52 (3.34, 3.72) | 4.34 (4.22, 4.47) | 4.04 (3.93, 4.15) | 4.49 (4.35, 4.63) | 4.03 (3.96, 4.11) |
| 80-84 | 3.48 (3.22, 3.76) | 3.89 (3.65, 4.14) | 4.20 (4.05, 4.35) | 3.65 (3.50, 3.81) | 5.05 (4.61, 5.53) | 4.49 (4.15, 4.86) | 2.87 (2.78, 2.96) | 3.25 (3.16, 3.35) | 3.73 (3.50, 3.98) | 2.99 (2.84, 3.16) | 3.92 (3.81, 4.03) | 3.93 (3.82, 4.03) | 4.58 (4.44, 4.73) | 4.39 (4.31, 4.47) |
| 85-89 | 4.83 (4.47, 5.22) | 3.95 (3.71, 4.21) | 4.64 (4.48, 4.81) | 4.54 (4.35, 4.74) | 5.44 (4.96, 5.96) | 4.66 (4.31, 5.04) | 2.57 (2.49, 2.65) | 2.94 (2.86, 3.03) | 2.13 (1.99, 2.27) | 2.03 (1.92, 2.14) | 3.91 (3.81, 4.03) | 4.30 (4.18, 4.42) | 5.03 (4.87, 5.19) | 5.04 (4.95, 5.14) |
| 90-94 | 4.82 (4.45, 5.22) | 3.26 (3.05, 3.47) | 3.59 (3.46, 3.72) | 4.57 (4.37, 4.77) | 4.44 (4.05, 4.88) | 4.24 (3.91, 4.59) | 2.29 (2.22, 2.37) | 2.36 (2.29, 2.43) | 1.74 (1.62, 1.86) | 2.09 (1.98, 2.21) | 2.93 (2.85, 3.01) | 3.47 (3.38, 3.57) | 4.07 (3.94, 4.21) | 4.82 (4.73, 4.91) |
| 95+ | 2.06 (1.89, 2.25) | 2.83 (2.64, 3.04) | 2.76 (2.65, 2.87) | 3.59 (3.42, 3.76) | 5.35 (4.84, 5.93) | 5.09 (4.66, 5.55) | 1.98 (1.91, 2.05) | 1.92 (1.86, 1.99) | 1.49 (1.38, 1.60) | 1.64 (1.55, 1.75) | 2.16 (2.09, 2.23) | 2.75 (2.67, 2.84) | 2.86 (2.76, 2.97) | 4.14 (4.06, 4.23) |
| **Period** | | | | | | | | | | | | | | |
| 1992-1996 | 0.59 (0.56, 0.61) | 0.72 (0.70, 0.75) | 0.67 (0.66, 0.68) | 0.68 (0.66, 0.69) | 0.55 (0.53, 0.58) | 0.60 (0.58, 0.63) | 0.73 (0.72, 0.74) | 0.74 (0.72, 0.75) | 0.69 (0.66, 0.71) | 0.78 (0.76, 0.81) | 0.72 (0.71, 0.73) | 0.71 (0.70, 0.72) | 0.66 (0.65, 0.67) | 0.73 (0.72, 0.74) |
| 1997-2001 | 0.69 (0.66, 0.72) | 0.81 (0.79, 0.84) | 0.79 (0.78, 0.81) | 0.79 (0.77, 0.80) | 0.73 (0.69, 0.76) | 0.74 (0.71, 0.78) | 0.80 (0.79, 0.81) | 0.81 (0.80, 0.83) | 0.75 (0.73, 0.78) | 0.84 (0.82, 0.87) | 0.85 (0.83, 0.86) | 0.86 (0.84, 0.87) | 0.77 (0.76, 0.79) | 0.83 (0.82, 0.84) |
| 2002-2006 | 0.86 (0.82, 0.89) | 0.90 (0.87, 0.93) | 0.91 (0.89, 0.92) | 0.91 (0.89, 0.93) | 0.92 (0.87, 0.96) | 0.92 (0.88, 0.96) | 0.92 (0.90, 0.93) | 0.92 (0.91, 0.94) | 0.90 (0.86, 0.93) | 0.88 (0.85, 0.90) | 0.97 (0.95, 0.98) | 0.98 (0.96, 0.99) | 0.91 (0.89, 0.92) | 0.93 (0.92, 0.94) |
| 2007-2011 | 1.10 (1.05, 1.14) | 1.04 (1.00, 1.07) | 1.08 (1.06, 1.10) | 1.07 (1.05, 1.10) | 1.22 (1.16, 1.28) | 1.21 (1.16, 1.26) | 1.10 (1.08, 1.12) | 1.10 (1.08, 1.12) | 1.08 (1.04, 1.12) | 1.04 (1.01, 1.07) | 1.08 (1.06, 1.09) | 1.08 (1.07, 1.10) | 1.08 (1.06, 1.10) | 1.06 (1.05, 1.07) |
| 2012-2016 | 1.41 (1.35, 1.47) | 1.20 (1.16, 1.24) | 1.31 (1.29, 1.34) | 1.31 (1.28, 1.34) | 1.40 (1.34, 1.47) | 1.33 (1.28, 1.39) | 1.23 (1.21, 1.25) | 1.21 (1.20, 1.23) | 1.28 (1.24, 1.33) | 1.21 (1.18, 1.25) | 1.20 (1.18, 1.22) | 1.20 (1.18, 1.21) | 1.28 (1.26, 1.31) | 1.21 (1.19, 1.22) |
| 2017-2021 | 1.87 (1.79, 1.95) | 1.52 (1.47, 1.57) | 1.46 (1.44, 1.49) | 1.48 (1.44, 1.51) | 1.59 (1.51, 1.67) | 1.51 (1.45, 1.58) | 1.38 (1.35, 1.40) | 1.36 (1.33, 1.38) | 1.55 (1.50, 1.61) | 1.38 (1.34, 1.42) | 1.32 (1.30, 1.34) | 1.31 (1.29, 1.33) | 1.55 (1.52, 1.58) | 1.40 (1.38, 1.41) |
| **Cohort** | | | | | | | | | | | | | | |
| 1897-1901 | 4.15 (3.45, 4.99) | 4.24 (3.66, 4.92) | 4.21 (3.87, 4.58) | 3.07 (2.77, 3.40) | 4.75 (3.83, 5.89) | 3.95 (3.28, 4.76) | 3.60 (3.34, 3.87) | 3.50 (3.26, 3.76) | 3.36 (2.88, 3.92) | 2.47 (2.18, 2.81) | 5.59 (5.23, 5.98) | 5.26 (4.94, 5.61) | 4.79 (4.44, 5.16) | 4.38 (4.19, 4.57) |
| 1902-1906 | 3.39 (2.97, 3.86) | 3.45 (3.11, 3.84) | 3.80 (3.58, 4.03) | 3.16 (2.94, 3.40) | 3.72 (3.19, 4.34) | 2.95 (2.58, 3.36) | 3.12 (2.96, 3.29) | 3.14 (2.98, 3.30) | 3.35 (3.00, 3.73) | 2.46 (2.25, 2.70) | 4.78 (4.55, 5.01) | 4.52 (4.32, 4.73) | 3.96 (3.76, 4.18) | 3.81 (3.69, 3.93) |
| 1907-1911 | 2.83 (2.54, 3.16) | 2.74 (2.51, 2.99) | 3.57 (3.40, 3.75) | 3.19 (3.01, 3.39) | 2.99 (2.63, 3.39) | 2.48 (2.22, 2.76) | 2.65 (2.54, 2.77) | 2.71 (2.60, 2.83) | 2.85 (2.60, 3.11) | 2.36 (2.19, 2.54) | 4.04 (3.89, 4.21) | 3.88 (3.73, 4.02) | 3.40 (3.26, 3.56) | 3.35 (3.26, 3.43) |
| 1912-1916 | 2.47 (2.25, 2.72) | 2.19 (2.03, 2.36) | 3.18 (3.05, 3.32) | 3.15 (2.99, 3.32) | 2.51 (2.25, 2.80) | 2.41 (2.19, 2.65) | 2.26 (2.17, 2.34) | 2.33 (2.25, 2.42) | 2.32 (2.15, 2.51) | 2.13 (1.99, 2.27) | 3.39 (3.28, 3.51) | 3.29 (3.18, 3.40) | 3.01 (2.90, 3.13) | 3.02 (2.96, 3.09) |
| 1917-1921 | 2.19 (2.01, 2.38) | 1.85 (1.73, 1.99) | 2.71 (2.61, 2.82) | 2.89 (2.75, 3.03) | 2.21 (2.00, 2.44) | 2.36 (2.17, 2.57) | 1.96 (1.89, 2.03) | 2.05 (1.99, 2.12) | 1.93 (1.80, 2.07) | 1.99 (1.88, 2.12) | 2.76 (2.68, 2.85) | 2.75 (2.67, 2.83) | 2.52 (2.44, 2.61) | 2.57 (2.52, 2.63) |
| 1922-1926 | 1.87 (1.73, 2.02) | 1.71 (1.60, 1.82) | 2.25 (2.17, 2.33) | 2.49 (2.39, 2.61) | 2.08 (1.90, 2.28) | 2.26 (2.08, 2.44) | 1.71 (1.66, 1.77) | 1.80 (1.75, 1.85) | 1.77 (1.65, 1.89) | 1.86 (1.76, 1.96) | 2.24 (2.18, 2.31) | 2.27 (2.21, 2.33) | 2.26 (2.19, 2.34) | 2.29 (2.24, 2.33) |
| 1927-1931 | 1.64 (1.51, 1.78) | 1.63 (1.52, 1.74) | 2.08 (2.01, 2.16) | 2.12 (2.03, 2.22) | 2.00 (1.82, 2.20) | 2.16 (1.99, 2.35) | 1.50 (1.46, 1.55) | 1.59 (1.54, 1.64) | 1.64 (1.53, 1.75) | 1.75 (1.66, 1.86) | 1.83 (1.77, 1.88) | 1.88 (1.83, 1.93) | 1.96 (1.90, 2.03) | 1.94 (1.90, 1.98) |
| 1932-1936 | 1.53 (1.40, 1.66) | 1.54 (1.44, 1.64) | 1.80 (1.73, 1.87) | 1.80 (1.72, 1.89) | 1.77 (1.60, 1.95) | 1.89 (1.74, 2.06) | 1.34 (1.30, 1.39) | 1.41 (1.37, 1.46) | 1.52 (1.41, 1.63) | 1.67 (1.57, 1.77) | 1.47 (1.42, 1.51) | 1.54 (1.50, 1.59) | 1.67 (1.61, 1.72) | 1.64 (1.61, 1.67) |
| 1937-1941 | 1.37 (1.26, 1.50) | 1.41 (1.32, 1.52) | 1.50 (1.44, 1.56) | 1.53 (1.46, 1.60) | 1.66 (1.50, 1.84) | 1.65 (1.51, 1.80) | 1.21 (1.17, 1.25) | 1.27 (1.23, 1.31) | 1.28 (1.19, 1.37) | 1.50 (1.41, 1.59) | 1.16 (1.12, 1.20) | 1.23 (1.19, 1.26) | 1.41 (1.36, 1.46) | 1.39 (1.36, 1.42) |
| 1942-1946 | 1.21 (1.11, 1.32) | 1.28 (1.19, 1.37) | 1.28 (1.23, 1.33) | 1.28 (1.22, 1.35) | 1.53 (1.38, 1.69) | 1.46 (1.34, 1.60) | 1.11 (1.07, 1.15) | 1.15 (1.11, 1.19) | 1.12 (1.04, 1.20) | 1.37 (1.29, 1.46) | 0.93 (0.90, 0.96) | 0.98 (0.95, 1.01) | 1.21 (1.16, 1.25) | 1.21 (1.19, 1.24) |
| 1947-1951 | 1.10 (1.01, 1.21) | 1.16 (1.08, 1.25) | 1.10 (1.05, 1.14) | 1.09 (1.04, 1.14) | 1.26 (1.13, 1.39) | 1.14 (1.04, 1.24) | 1.02 (0.98, 1.05) | 1.06 (1.02, 1.09) | 1.00 (0.93, 1.08) | 1.22 (1.14, 1.29) | 0.77 (0.74, 0.79) | 0.80 (0.77, 0.82) | 1.05 (1.02, 1.09) | 1.06 (1.04, 1.08) |
| 1952-1956 | 0.97 (0.89, 1.06) | 1.04 (0.97, 1.12) | 0.99 (0.95, 1.03) | 0.98 (0.94, 1.03) | 1.14 (1.03, 1.27) | 0.95 (0.87, 1.04) | 0.92 (0.89, 0.95) | 0.94 (0.91, 0.97) | 0.90 (0.83, 0.96) | 1.06 (0.99, 1.12) | 0.70 (0.68, 0.73) | 0.70 (0.68, 0.72) | 0.93 (0.89, 0.96) | 0.93 (0.91, 0.95) |
| 1957-1961 | 0.86 (0.79, 0.94) | 0.92 (0.86, 0.99) | 0.79 (0.76, 0.82) | 0.80 (0.76, 0.84) | 1.01 (0.91, 1.12) | 0.82 (0.75, 0.90) | 0.85 (0.82, 0.88) | 0.85 (0.83, 0.88) | 0.79 (0.73, 0.84) | 0.86 (0.81, 0.92) | 0.65 (0.63, 0.67) | 0.65 (0.63, 0.67) | 0.80 (0.77, 0.83) | 0.81 (0.79, 0.83) |
| 1962-1966 | 0.74 (0.68, 0.81) | 0.78 (0.73, 0.83) | 0.66 (0.63, 0.69) | 0.68 (0.65, 0.72) | 0.90 (0.82, 1.00) | 0.76 (0.69, 0.82) | 0.77 (0.74, 0.80) | 0.77 (0.74, 0.79) | 0.70 (0.66, 0.76) | 0.76 (0.72, 0.81) | 0.60 (0.58, 0.62) | 0.61 (0.59, 0.63) | 0.69 (0.66, 0.71) | 0.70 (0.69, 0.72) |
| 1967-1971 | 0.67 (0.62, 0.73) | 0.68 (0.64, 0.73) | 0.57 (0.55, 0.59) | 0.59 (0.56, 0.62) | 0.73 (0.66, 0.81) | 0.65 (0.60, 0.71) | 0.70 (0.67, 0.72) | 0.69 (0.67, 0.71) | 0.66 (0.61, 0.71) | 0.67 (0.63, 0.71) | 0.55 (0.54, 0.57) | 0.56 (0.55, 0.58) | 0.62 (0.60, 0.64) | 0.61 (0.60, 0.62) |
| 1972-1976 | 0.59 (0.55, 0.64) | 0.59 (0.55, 0.63) | 0.48 (0.46, 0.50) | 0.50 (0.48, 0.53) | 0.59 (0.54, 0.65) | 0.57 (0.53, 0.62) | 0.63 (0.61, 0.65) | 0.62 (0.60, 0.64) | 0.61 (0.57, 0.66) | 0.59 (0.56, 0.62) | 0.52 (0.50, 0.53) | 0.52 (0.50, 0.53) | 0.54 (0.52, 0.56) | 0.53 (0.52, 0.54) |
| 1977-1981 | 0.55 (0.51, 0.60) | 0.53 (0.50, 0.56) | 0.43 (0.42, 0.45) | 0.45 (0.43, 0.47) | 0.48 (0.44, 0.53) | 0.51 (0.47, 0.55) | 0.56 (0.55, 0.58) | 0.56 (0.54, 0.57) | 0.55 (0.52, 0.59) | 0.53 (0.50, 0.56) | 0.51 (0.50, 0.52) | 0.51 (0.49, 0.52) | 0.47 (0.46, 0.49) | 0.47 (0.46, 0.47) |
| 1982-1986 | 0.49 (0.45, 0.53) | 0.46 (0.43, 0.50) | 0.38 (0.37, 0.40) | 0.39 (0.38, 0.41) | 0.37 (0.34, 0.41) | 0.43 (0.40, 0.47) | 0.51 (0.49, 0.52) | 0.49 (0.48, 0.51) | 0.47 (0.43, 0.50) | 0.43 (0.41, 0.46) | 0.48 (0.47, 0.50) | 0.48 (0.47, 0.49) | 0.41 (0.39, 0.42) | 0.41 (0.40, 0.42) |
| 1987-1991 | 0.41 (0.37, 0.45) | 0.39 (0.37, 0.42) | 0.34 (0.33, 0.36) | 0.36 (0.34, 0.37) | 0.28 (0.25, 0.31) | 0.33 (0.30, 0.36) | 0.46 (0.44, 0.48) | 0.44 (0.42, 0.45) | 0.41 (0.38, 0.44) | 0.39 (0.36, 0.41) | 0.44 (0.42, 0.45) | 0.43 (0.42, 0.44) | 0.36 (0.35, 0.38) | 0.36 (0.35, 0.37) |
| 1992-1996 | 0.33 (0.30, 0.37) | 0.34 (0.32, 0.37) | 0.30 (0.28, 0.31) | 0.30 (0.28, 0.32) | 0.23 (0.20, 0.26) | 0.27 (0.24, 0.30) | 0.42 (0.40, 0.43) | 0.39 (0.37, 0.40) | 0.40 (0.37, 0.44) | 0.36 (0.33, 0.38) | 0.36 (0.35, 0.38) | 0.37 (0.36, 0.38) | 0.30 (0.28, 0.31) | 0.30 (0.29, 0.31) |
| 1997-2001 | 0.28 (0.24, 0.32) | 0.29 (0.27, 0.33) | 0.24 (0.23, 0.26) | 0.25 (0.23, 0.27) | 0.18 (0.16, 0.21) | 0.25 (0.22, 0.28) | 0.37 (0.35, 0.39) | 0.33 (0.32, 0.35) | 0.38 (0.34, 0.43) | 0.37 (0.34, 0.41) | 0.27 (0.26, 0.28) | 0.29 (0.28, 0.30) | 0.24 (0.22, 0.25) | 0.25 (0.24, 0.25) |
| 2002-2006 | 0.21 (0.17, 0.26) | 0.24 (0.20, 0.28) | 0.20 (0.18, 0.22) | 0.24 (0.21, 0.26) | 0.16 (0.12, 0.20) | 0.22 (0.18, 0.27) | 0.30 (0.28, 0.33) | 0.27 (0.25, 0.30) | 0.34 (0.29, 0.40) | 0.37 (0.32, 0.43) | 0.24 (0.22, 0.25) | 0.21 (0.20, 0.23) | 0.19 (0.17, 0.20) | 0.20 (0.19, 0.21) |

RR: relative risk; CI: confidence interval.
