# Supplementary material for: Trends in incidence, mortality and disability-adjusted life years of colorectal cancer in East Asia (1990–2021): An analysis of the Global Burden of Disease study 2021
Source: PLoS One. 2025 Oct 8;20(10):e0334229. doi: 10.1371/journal.pone.0334229 (PMC12507298; doi:10.1371/journal.pone.0334229)
Supplement: S5 Table — (DOCX) [file pone.0334229.s005.docx]

**S5 Table.** **Age-, period-, and cohort-specific relative risks of CRC mortality** **for males and females in five East Asian countries, the United States, and globally, based on the age-period-cohort model**

|  | **China** | | | | **Japan** | | | | **South Korea** | | | | **North Korea** | | | **Mongolia** | | | | **United States** | | | | **Global** | | | |  |
| --- | --- | --- | --- | --- | --- | --- | --- | --- | --- | --- | --- | --- | --- | --- | --- | --- | --- | --- | --- | --- | --- | --- | --- | --- | --- | --- | --- | --- |
|  | **Male** | | **Female** | | **Male** | | **Female** | | **Male** | | **Female** | | **Male** | **Female** | | **Male** | | **Female** | | **Male** | | **Female** | | **Male** | | **Female** | |  |
|  | **RR (95% CI)** | | **RR (95% CI)** | | **RR (95% CI)** | | **RR (95% CI)** | | **RR (95% CI)** | | **RR (95% CI)** | | **RR (95% CI)** | **RR (95% CI)** | | **RR (95% CI)** | | **RR (95% CI)** | | **RR (95% CI)** | | **RR (95% CI)** | | **RR (95% CI)** | | **RR (95% CI)** | |  |
| **Age** | | | | | | | | | | | | | | | | | | | | | | | | | | | |  |
| 15-19 | | 0.06 (0.05, 0.06) | | 0.06 (0.06, 0.07) | | 0.03 (0.03, 0.03) | | 0.03 (0.03, 0.03) | | 0.04 (0.03, 0.04) | | 0.04 (0.03, 0.04) | 0.07 (0.06, 0.07) | | 0.07 (0.06, 0.07) | | 0.04 (0.04, 0.05) | | 0.05 (0.05, 0.05) | | 0.02 (0.02, 0.02) | | 0.02 (0.02, 0.02) | | 0.04 (0.04, 0.04) | | 0.04 (0.04, 0.04) | |
| 20-24 | | 0.09 (0.08, 0.09) | | 0.10 (0.09, 0.10) | | 0.05 (0.05, 0.05) | | 0.06 (0.05, 0.06) | | 0.05 (0.05, 0.06) | | 0.07 (0.06, 0.07) | 0.10 (0.10, 0.11) | | 0.10 (0.10, 0.10) | | 0.08 (0.08, 0.09) | | 0.09 (0.09, 0.10) | | 0.04 (0.04, 0.04) | | 0.04 (0.04, 0.04) | | 0.07 (0.06, 0.07) | | 0.08 (0.07, 0.08) | |
| 25-29 | | 0.14 (0.13, 0.15) | | 0.14 (0.14, 0.15) | | 0.08 (0.08, 0.08) | | 0.10 (0.10, 0.10) | | 0.10 (0.09, 0.11) | | 0.12 (0.11, 0.13) | 0.14 (0.14, 0.15) | | 0.13 (0.13, 0.14) | | 0.15 (0.14, 0.16) | | 0.18 (0.17, 0.18) | | 0.08 (0.08, 0.08) | | 0.09 (0.09, 0.09) | | 0.10 (0.10, 0.11) | | 0.11 (0.11, 0.11) | |
| 30-34 | | 0.25 (0.24, 0.27) | | 0.24 (0.23, 0.25) | | 0.16 (0.16, 0.16) | | 0.19 (0.18, 0.20) | | 0.17 (0.16, 0.19) | | 0.22 (0.20, 0.24) | 0.25 (0.24, 0.25) | | 0.23 (0.22, 0.23) | | 0.28 (0.26, 0.29) | | 0.28 (0.26, 0.29) | | 0.16 (0.16, 0.17) | | 0.18 (0.17, 0.18) | | 0.19 (0.19, 0.20) | | 0.19 (0.19, 0.19) | |
| 35-39 | | 0.38 (0.35, 0.41) | | 0.35 (0.34, 0.37) | | 0.26 (0.25, 0.26) | | 0.32 (0.31, 0.33) | | 0.25 (0.22, 0.27) | | 0.33 (0.30, 0.35) | 0.37 (0.36, 0.38) | | 0.35 (0.34, 0.36) | | 0.39 (0.37, 0.42) | | 0.42 (0.39, 0.44) | | 0.29 (0.28, 0.30) | | 0.32 (0.31, 0.33) | | 0.30 (0.29, 0.31) | | 0.29 (0.29, 0.30) | |
| 40-44 | | 0.52 (0.48, 0.56) | | 0.49 (0.46, 0.51) | | 0.43 (0.42, 0.44) | | 0.51 (0.49, 0.54) | | 0.38 (0.35, 0.42) | | 0.49 (0.46, 0.53) | 0.53 (0.52, 0.55) | | 0.55 (0.53, 0.56) | | 0.59 (0.55, 0.63) | | 0.65 (0.61, 0.68) | | 0.53 (0.51, 0.54) | | 0.57 (0.55, 0.58) | | 0.44 (0.43, 0.45) | | 0.45 (0.44, 0.45) | |
| 45-49 | | 0.69 (0.64, 0.75) | | 0.59 (0.56, 0.62) | | 0.71 (0.70, 0.73) | | 0.81 (0.77, 0.84) | | 0.59 (0.53, 0.64) | | 0.73 (0.68, 0.80) | 0.87 (0.85, 0.90) | | 0.83 (0.81, 0.85) | | 0.92 (0.86, 0.98) | | 0.94 (0.89, 1.00) | | 0.96 (0.93, 0.99) | | 0.97 (0.94, 0.99) | | 0.68 (0.67, 0.70) | | 0.69 (0.68, 0.70) | |
| 50-54 | | 0.94 (0.87, 1.01) | | 0.90 (0.85, 0.95) | | 1.18 (1.15, 1.22) | | 1.18 (1.13, 1.24) | | 0.92 (0.83, 1.01) | | 0.96 (0.88, 1.04) | 1.23 (1.19, 1.26) | | 1.21 (1.18, 1.24) | | 1.39 (1.30, 1.49) | | 1.33 (1.25, 1.40) | | 1.57 (1.53, 1.62) | | 1.45 (1.41, 1.49) | | 1.06 (1.03, 1.09) | | 1.04 (1.03, 1.06) | |
| 55-59 | | 1.25 (1.16, 1.35) | | 1.22 (1.16, 1.29) | | 1.79 (1.74, 1.84) | | 1.52 (1.46, 1.59) | | 1.32 (1.20, 1.45) | | 1.19 (1.09, 1.29) | 1.66 (1.62, 1.71) | | 1.60 (1.55, 1.64) | | 1.93 (1.80, 2.06) | | 1.72 (1.63, 1.82) | | 2.24 (2.18, 2.31) | | 1.92 (1.87, 1.97) | | 1.53 (1.49, 1.58) | | 1.41 (1.39, 1.43) | |
| 60-64 | | 1.54 (1.43, 1.67) | | 1.55 (1.46, 1.64) | | 2.49 (2.42, 2.56) | | 1.85 (1.77, 1.93) | | 1.85 (1.68, 2.04) | | 1.41 (1.30, 1.53) | 2.14 (2.08, 2.21) | | 2.04 (1.98, 2.09) | | 2.58 (2.41, 2.76) | | 2.35 (2.22, 2.48) | | 2.93 (2.84, 3.02) | | 2.39 (2.32, 2.46) | | 2.11 (2.05, 2.17) | | 1.80 (1.77, 1.83) | |
| 65-69 | | 1.98 (1.84, 2.14) | | 2.05 (1.94, 2.16) | | 3.17 (3.09, 3.26) | | 2.16 (2.07, 2.25) | | 2.43 (2.21, 2.67) | | 1.79 (1.65, 1.94) | 2.67 (2.59, 2.74) | | 2.56 (2.49, 2.63) | | 3.36 (3.14, 3.59) | | 3.00 (2.83, 3.17) | | 3.47 (3.37, 3.58) | | 2.83 (2.75, 2.91) | | 2.75 (2.67, 2.82) | | 2.29 (2.25, 2.33) | |
| 70-74 | | 2.68 (2.49, 2.88) | | 2.96 (2.81, 3.12) | | 3.82 (3.72, 3.92) | | 2.63 (2.52, 2.74) | | 3.31 (3.02, 3.63) | | 2.46 (2.27, 2.66) | 3.14 (3.05, 3.23) | | 3.29 (3.20, 3.37) | | 3.74 (3.50, 3.99) | | 3.12 (2.96, 3.30) | | 3.93 (3.82, 4.05) | | 3.34 (3.25, 3.43) | | 3.47 (3.38, 3.56) | | 2.99 (2.95, 3.04) | |
| 75-79 | | 3.47 (3.22, 3.73) | | 3.86 (3.66, 4.07) | | 4.51 (4.40, 4.63) | | 3.43 (3.29, 3.57) | | 4.58 (4.18, 5.01) | | 3.67 (3.39, 3.96) | 3.57 (3.47, 3.67) | | 3.86 (3.76, 3.96) | | 4.24 (3.97, 4.52) | | 3.81 (3.61, 4.02) | | 4.42 (4.30, 4.55) | | 4.15 (4.04, 4.26) | | 4.28 (4.17, 4.39) | | 3.89 (3.83, 3.95) | |
| 80-84 | | 4.17 (3.88, 4.49) | | 4.78 (4.54, 5.04) | | 5.54 (5.40, 5.69) | | 4.72 (4.53, 4.92) | | 6.27 (5.73, 6.86) | | 5.51 (5.10, 5.95) | 3.68 (3.58, 3.78) | | 4.14 (4.04, 4.25) | | 4.48 (4.21, 4.78) | | 3.66 (3.47, 3.86) | | 4.92 (4.79, 5.06) | | 4.95 (4.82, 5.08) | | 5.21 (5.08, 5.35) | | 5.01 (4.93, 5.08) | |
| 85-89 | | 6.78 (6.30, 7.29) | | 5.68 (5.39, 5.99) | | 7.10 (6.92, 7.28) | | 7.03 (6.74, 7.32) | | 7.78 (7.11, 8.51) | | 6.71 (6.21, 7.25) | 3.66 (3.56, 3.76) | | 4.18 (4.07, 4.28) | | 2.80 (2.63, 2.99) | | 2.72 (2.58, 2.87) | | 5.51 (5.36, 5.67) | | 6.06 (5.91, 6.22) | | 6.48 (6.31, 6.65) | | 6.41 (6.31, 6.50) | |
| 90-94 | | 9.01 (8.37, 9.70) | | 6.26 (5.93, 6.60) | | 8.43 (8.21, 8.65) | | 10.09 (9.68, 10.52) | | 9.33 (8.51, 10.23) | | 8.94 (8.26, 9.67) | 3.77 (3.66, 3.88) | | 3.92 (3.82, 4.03) | | 2.62 (2.45, 2.79) | | 3.21 (3.04, 3.39) | | 6.10 (5.93, 6.28) | | 7.13 (6.94, 7.32) | | 7.30 (7.10, 7.49) | | 8.31 (8.18, 8.44) | |
| 95+ | | 4.75 (4.38, 5.15) | | 6.86 (6.47, 7.27) | | 7.37 (7.16, 7.58) | | 11.10 (10.60, 11.61) | | 14.72 (13.31, 16.28) | | 14.57 (13.37, 15.87) | 3.65 (3.54, 3.77) | | 3.63 (3.53, 3.74) | | 2.52 (2.35, 2.70) | | 2.84 (2.68, 3.02) | | 5.95 (5.77, 6.14) | | 7.66 (7.44, 7.89) | | 6.25 (6.07, 6.44) | | 9.33 (9.17, 9.49) | |
| **Period** | | | | | | | | | | | | | | | | | | | | | | | | | | | |  |
| 1992-1996 | | 0.74 (0.72, 0.77) | | 0.93 (0.90, 0.96) | | 0.75 (0.74, 0.76) | | 0.77 (0.75, 0.78) | | 0.80 (0.76, 0.84) | | 0.86 (0.83, 0.90) | 0.76 (0.75, 0.77) | | 0.77 (0.76, 0.78) | | 0.71 (0.69, 0.74) | | 0.82 (0.79, 0.84) | | 0.77 (0.76, 0.78) | | 0.76 (0.75, 0.77) | | 0.75 (0.74, 0.76) | | 0.82 (0.81, 0.82) | |
| 1997-2001 | | 0.81 (0.78, 0.85) | | 0.97 (0.94, 1.00) | | 0.83 (0.82, 0.84) | | 0.83 (0.82, 0.85) | | 0.88 (0.84, 0.93) | | 0.90 (0.86, 0.94) | 0.83 (0.82, 0.84) | | 0.85 (0.84, 0.86) | | 0.79 (0.76, 0.82) | | 0.88 (0.86, 0.91) | | 0.85 (0.84, 0.86) | | 0.86 (0.85, 0.88) | | 0.83 (0.82, 0.84) | | 0.89 (0.88, 0.89) | |
| 2002-2006 | | 0.92 (0.88, 0.95) | | 0.97 (0.94, 0.99) | | 0.92 (0.91, 0.94) | | 0.93 (0.91, 0.95) | | 0.95 (0.90, 0.99) | | 0.95 (0.91, 0.99) | 0.93 (0.92, 0.95) | | 0.94 (0.93, 0.95) | | 0.92 (0.89, 0.95) | | 0.90 (0.87, 0.92) | | 0.95 (0.94, 0.97) | | 0.96 (0.95, 0.97) | | 0.93 (0.92, 0.94) | | 0.95 (0.94, 0.95) | |
| 2007-2011 | | 1.02 (0.98, 1.06) | | 0.96 (0.93, 0.99) | | 1.04 (1.02, 1.05) | | 1.02 (1.00, 1.04) | | 1.05 (1.00, 1.10) | | 1.04 (1.00, 1.09) | 1.08 (1.07, 1.10) | | 1.08 (1.07, 1.10) | | 1.06 (1.02, 1.10) | | 1.01 (0.99, 1.04) | | 1.04 (1.03, 1.06) | | 1.04 (1.03, 1.06) | | 1.04 (1.02, 1.05) | | 1.01 (1.00, 1.02) | |
| 2012-2016 | | 1.20 (1.15, 1.25) | | 1.01 (0.98, 1.04) | | 1.23 (1.21, 1.24) | | 1.21 (1.18, 1.24) | | 1.13 (1.08, 1.19) | | 1.08 (1.04, 1.12) | 1.20 (1.18, 1.22) | | 1.18 (1.16, 1.19) | | 1.24 (1.20, 1.29) | | 1.17 (1.14, 1.21) | | 1.17 (1.15, 1.19) | | 1.17 (1.15, 1.18) | | 1.19 (1.18, 1.21) | | 1.12 (1.11, 1.13) | |
| 2017-2021 | | 1.47 (1.42, 1.53) | | 1.19 (1.15, 1.22) | | 1.36 (1.34, 1.38) | | 1.37 (1.34, 1.40) | | 1.26 (1.20, 1.33) | | 1.21 (1.16, 1.26) | 1.32 (1.30, 1.34) | | 1.29 (1.27, 1.31) | | 1.47 (1.42, 1.52) | | 1.30 (1.26, 1.34) | | 1.31 (1.29, 1.33) | | 1.30 (1.28, 1.32) | | 1.40 (1.38, 1.42) | | 1.28 (1.27, 1.29) | |
| **Cohort** | | | | | | | | | | | | | | | | | | | | | | | | | | | |  |
| 1897-1901 | | 3.75 (3.16, 4.46) | | 3.79 (3.34, 4.29) | | 4.62 (4.35, 4.90) | | 3.22 (2.93, 3.55) | | 4.21 (3.40, 5.21) | | 3.36 (2.80, 4.03) | 3.86 (3.61, 4.11) | | 3.72 (3.50, 3.95) | | 3.48 (3.00, 4.05) | | 2.56 (2.25, 2.90) | | 6.36 (5.96, 6.80) | | 5.94 (5.59, 6.31) | | 4.64 (4.37, 4.94) | | 4.21 (4.06, 4.36) | |
| 1902-1906 | | 3.28 (2.90, 3.70) | | 3.33 (3.04, 3.63) | | 4.23 (4.06, 4.42) | | 3.40 (3.17, 3.64) | | 3.68 (3.16, 4.29) | | 2.86 (2.51, 3.26) | 3.37 (3.22, 3.53) | | 3.36 (3.22, 3.51) | | 3.48 (3.13, 3.88) | | 2.56 (2.34, 2.80) | | 5.54 (5.28, 5.80) | | 5.22 (5.00, 5.45) | | 3.96 (3.79, 4.14) | | 3.81 (3.72, 3.91) | |
| 1907-1911 | | 2.98 (2.69, 3.29) | | 2.88 (2.68, 3.10) | | 4.03 (3.89, 4.17) | | 3.52 (3.32, 3.72) | | 3.28 (2.89, 3.72) | | 2.70 (2.42, 3.00) | 2.90 (2.79, 3.01) | | 2.96 (2.86, 3.07) | | 3.01 (2.76, 3.29) | | 2.49 (2.31, 2.68) | | 4.73 (4.55, 4.91) | | 4.53 (4.37, 4.70) | | 3.50 (3.38, 3.63) | | 3.45 (3.38, 3.53) | |
| 1912-1916 | | 2.79 (2.55, 3.04) | | 2.48 (2.32, 2.64) | | 3.66 (3.55, 3.78) | | 3.56 (3.39, 3.74) | | 2.95 (2.65, 3.29) | | 2.83 (2.58, 3.11) | 2.51 (2.42, 2.59) | | 2.59 (2.51, 2.67) | | 2.50 (2.32, 2.70) | | 2.30 (2.15, 2.45) | | 3.95 (3.82, 4.09) | | 3.86 (3.74, 3.98) | | 3.17 (3.08, 3.28) | | 3.20 (3.14, 3.26) | |
| 1917-1921 | | 2.59 (2.39, 2.80) | | 2.21 (2.08, 2.34) | | 3.14 (3.05, 3.23) | | 3.33 (3.19, 3.48) | | 2.70 (2.44, 2.97) | | 2.90 (2.67, 3.15) | 2.20 (2.13, 2.26) | | 2.30 (2.24, 2.37) | | 2.11 (1.97, 2.26) | | 2.19 (2.06, 2.32) | | 3.19 (3.10, 3.29) | | 3.20 (3.11, 3.29) | | 2.73 (2.65, 2.81) | | 2.77 (2.72, 2.82) | |
| 1922-1926 | | 2.26 (2.10, 2.43) | | 2.08 (1.98, 2.20) | | 2.58 (2.51, 2.65) | | 2.87 (2.75, 2.99) | | 2.57 (2.35, 2.81) | | 2.80 (2.59, 3.03) | 1.93 (1.88, 1.98) | | 2.03 (1.98, 2.08) | | 1.95 (1.83, 2.08) | | 2.06 (1.95, 2.18) | | 2.56 (2.49, 2.64) | | 2.61 (2.54, 2.68) | | 2.48 (2.41, 2.54) | | 2.49 (2.46, 2.53) | |
| 1927-1931 | | 1.97 (1.83, 2.13) | | 1.99 (1.88, 2.10) | | 2.33 (2.27, 2.39) | | 2.42 (2.32, 2.52) | | 2.45 (2.23, 2.70) | | 2.68 (2.47, 2.91) | 1.69 (1.64, 1.74) | | 1.79 (1.74, 1.84) | | 1.81 (1.70, 1.94) | | 1.96 (1.85, 2.07) | | 2.06 (2.00, 2.12) | | 2.14 (2.08, 2.20) | | 2.16 (2.10, 2.22) | | 2.16 (2.13, 2.19) | |
| 1932-1936 | | 1.80 (1.67, 1.95) | | 1.85 (1.75, 1.95) | | 2.01 (1.95, 2.06) | | 2.03 (1.94, 2.12) | | 2.12 (1.93, 2.34) | | 2.30 (2.12, 2.50) | 1.49 (1.45, 1.54) | | 1.58 (1.53, 1.62) | | 1.67 (1.56, 1.79) | | 1.86 (1.76, 1.97) | | 1.63 (1.58, 1.68) | | 1.72 (1.68, 1.77) | | 1.84 (1.79, 1.90) | | 1.83 (1.80, 1.86) | |
| 1937-1941 | | 1.60 (1.48, 1.73) | | 1.68 (1.59, 1.78) | | 1.66 (1.61, 1.71) | | 1.70 (1.62, 1.78) | | 1.95 (1.77, 2.16) | | 1.95 (1.79, 2.13) | 1.32 (1.29, 1.37) | | 1.39 (1.35, 1.43) | | 1.40 (1.31, 1.50) | | 1.66 (1.56, 1.76) | | 1.26 (1.22, 1.30) | | 1.35 (1.31, 1.39) | | 1.55 (1.51, 1.60) | | 1.55 (1.53, 1.58) | |
| 1942-1946 | | 1.38 (1.27, 1.50) | | 1.49 (1.40, 1.58) | | 1.40 (1.36, 1.44) | | 1.41 (1.35, 1.48) | | 1.76 (1.59, 1.95) | | 1.68 (1.54, 1.84) | 1.20 (1.16, 1.23) | | 1.24 (1.20, 1.27) | | 1.21 (1.12, 1.30) | | 1.50 (1.41, 1.60) | | 0.99 (0.96, 1.02) | | 1.05 (1.02, 1.08) | | 1.31 (1.27, 1.34) | | 1.32 (1.30, 1.34) | |
| 1947-1951 | | 1.21 (1.11, 1.31) | | 1.31 (1.23, 1.39) | | 1.19 (1.16, 1.22) | | 1.20 (1.15, 1.26) | | 1.40 (1.26, 1.55) | | 1.27 (1.16, 1.38) | 1.07 (1.04, 1.10) | | 1.11 (1.08, 1.15) | | 1.06 (0.99, 1.14) | | 1.31 (1.23, 1.39) | | 0.80 (0.77, 0.83) | | 0.83 (0.81, 0.86) | | 1.12 (1.08, 1.15) | | 1.13 (1.11, 1.15) | |
| 1952-1956 | | 1.03 (0.95, 1.12) | | 1.12 (1.06, 1.19) | | 1.07 (1.03, 1.10) | | 1.07 (1.02, 1.12) | | 1.24 (1.11, 1.37) | | 1.02 (0.94, 1.12) | 0.95 (0.92, 0.98) | | 0.97 (0.94, 0.99) | | 0.93 (0.87, 1.00) | | 1.12 (1.05, 1.19) | | 0.71 (0.69, 0.74) | | 0.72 (0.70, 0.74) | | 0.98 (0.95, 1.01) | | 0.98 (0.96, 1.00) | |
| 1957-1961 | | 0.88 (0.81, 0.96) | | 0.96 (0.90, 1.01) | | 0.84 (0.81, 0.86) | | 0.84 (0.81, 0.88) | | 1.05 (0.95, 1.16) | | 0.85 (0.78, 0.93) | 0.85 (0.82, 0.87) | | 0.86 (0.83, 0.88) | | 0.80 (0.75, 0.86) | | 0.89 (0.84, 0.95) | | 0.64 (0.62, 0.66) | | 0.64 (0.63, 0.66) | | 0.83 (0.80, 0.85) | | 0.84 (0.82, 0.85) | |
| 1962-1966 | | 0.73 (0.68, 0.79) | | 0.78 (0.73, 0.82) | | 0.67 (0.65, 0.69) | | 0.69 (0.66, 0.73) | | 0.91 (0.83, 1.01) | | 0.76 (0.70, 0.83) | 0.75 (0.73, 0.78) | | 0.75 (0.73, 0.78) | | 0.70 (0.66, 0.76) | | 0.77 (0.73, 0.82) | | 0.58 (0.56, 0.60) | | 0.59 (0.57, 0.60) | | 0.70 (0.68, 0.72) | | 0.71 (0.70, 0.72) | |
| 1967-1971 | | 0.65 (0.60, 0.70) | | 0.67 (0.63, 0.70) | | 0.56 (0.54, 0.57) | | 0.58 (0.55, 0.60) | | 0.71 (0.65, 0.78) | | 0.63 (0.58, 0.68) | 0.67 (0.65, 0.69) | | 0.66 (0.64, 0.68) | | 0.64 (0.60, 0.69) | | 0.66 (0.62, 0.70) | | 0.52 (0.51, 0.54) | | 0.53 (0.52, 0.54) | | 0.62 (0.60, 0.64) | | 0.61 (0.60, 0.62) | |
| 1972-1976 | | 0.56 (0.52, 0.60) | | 0.56 (0.53, 0.59) | | 0.46 (0.45, 0.48) | | 0.48 (0.46, 0.50) | | 0.56 (0.51, 0.61) | | 0.54 (0.49, 0.58) | 0.59 (0.58, 0.61) | | 0.58 (0.57, 0.60) | | 0.59 (0.55, 0.63) | | 0.57 (0.54, 0.60) | | 0.48 (0.46, 0.49) | | 0.47 (0.46, 0.49) | | 0.53 (0.51, 0.54) | | 0.52 (0.51, 0.53) | |
| 1977-1981 | | 0.50 (0.47, 0.54) | | 0.48 (0.46, 0.50) | | 0.40 (0.39, 0.41) | | 0.41 (0.39, 0.43) | | 0.43 (0.40, 0.47) | | 0.46 (0.42, 0.49) | 0.52 (0.50, 0.53) | | 0.51 (0.50, 0.52) | | 0.51 (0.48, 0.55) | | 0.49 (0.47, 0.52) | | 0.46 (0.44, 0.47) | | 0.45 (0.44, 0.46) | | 0.45 (0.43, 0.46) | | 0.44 (0.43, 0.45) | |
| 1982-1986 | | 0.43 (0.39, 0.46) | | 0.40 (0.38, 0.42) | | 0.33 (0.33, 0.34) | | 0.35 (0.33, 0.36) | | 0.32 (0.29, 0.35) | | 0.37 (0.34, 0.40) | 0.45 (0.44, 0.47) | | 0.44 (0.43, 0.45) | | 0.42 (0.40, 0.45) | | 0.39 (0.37, 0.42) | | 0.42 (0.40, 0.43) | | 0.41 (0.40, 0.43) | | 0.37 (0.36, 0.38) | | 0.37 (0.36, 0.38) | |
| 1987-1991 | | 0.35 (0.32, 0.38) | | 0.32 (0.31, 0.35) | | 0.28 (0.27, 0.29) | | 0.29 (0.28, 0.31) | | 0.23 (0.20, 0.25) | | 0.27 (0.25, 0.30) | 0.40 (0.39, 0.41) | | 0.38 (0.37, 0.39) | | 0.36 (0.34, 0.39) | | 0.34 (0.32, 0.36) | | 0.37 (0.36, 0.38) | | 0.36 (0.35, 0.37) | | 0.32 (0.31, 0.33) | | 0.31 (0.31, 0.32) | |
| 1992-1996 | | 0.27 (0.25, 0.30) | | 0.27 (0.25, 0.29) | | 0.23 (0.22, 0.24) | | 0.24 (0.22, 0.25) | | 0.18 (0.16, 0.20) | | 0.21 (0.19, 0.23) | 0.35 (0.34, 0.37) | | 0.33 (0.32, 0.34) | | 0.35 (0.32, 0.38) | | 0.30 (0.28, 0.32) | | 0.30 (0.29, 0.31) | | 0.30 (0.29, 0.31) | | 0.26 (0.25, 0.27) | | 0.26 (0.25, 0.26) | |
| 1997-2001 | | 0.22 (0.20, 0.25) | | 0.22 (0.21, 0.24) | | 0.18 (0.17, 0.19) | | 0.19 (0.17, 0.20) | | 0.14 (0.12, 0.16) | | 0.19 (0.17, 0.21) | 0.30 (0.29, 0.32) | | 0.28 (0.26, 0.29) | | 0.32 (0.29, 0.36) | | 0.30 (0.28, 0.33) | | 0.22 (0.21, 0.23) | | 0.23 (0.22, 0.24) | | 0.20 (0.19, 0.21) | | 0.21 (0.21, 0.22) | |
| 2002-2006 | | 0.16 (0.13, 0.19) | | 0.17 (0.15, 0.20) | | 0.14 (0.13, 0.15) | | 0.17 (0.15, 0.19) | | 0.11 (0.09, 0.14) | | 0.16 (0.13, 0.20) | 0.25 (0.23, 0.26) | | 0.22 (0.21, 0.23) | | 0.28 (0.23, 0.33) | | 0.29 (0.25, 0.34) | | 0.19 (0.17, 0.20) | | 0.16 (0.15, 0.18) | | 0.16 (0.15, 0.17) | | 0.17 (0.17, 0.18) | |

RR: relative risk; CI: confidence interval.
