# Supplementary material for: Trends in incidence, mortality and disability-adjusted life years of colorectal cancer in East Asia (1990–2021): An analysis of the Global Burden of Disease study 2021
Source: PLoS One. 2025 Oct 8;20(10):e0334229. doi: 10.1371/journal.pone.0334229 (PMC12507298; doi:10.1371/journal.pone.0334229)
Supplement: S6 Table — (DOCX) [file pone.0334229.s006.docx]

**S6 Table.** **Age-, period-, and cohort-specific relative risks of CRC DALYs for males and females in five East Asian countries, the United States, and globally, based on the age-period-cohort model**

|  | **China** | | **Japan** | | **South Korea** | | **North Korea** | | **Mongolia** | | **United States** | | **Global** | |  |
| --- | --- | --- | --- | --- | --- | --- | --- | --- | --- | --- | --- | --- | --- | --- | --- |
|  | **Male** | **Female** | **Male** | **Female** | **Male** | **Female** | **Male** | **Female** | **Male** | **Female** | **Male** | **Female** | **Male** | **Female** |  |
|  | **RR (95% CI)** | **RR (95% CI)** | **RR (95% CI)** | **RR (95% CI)** | **RR (95% CI)** | **RR (95% CI)** | **RR (95% CI)** | **RR (95% CI)** | **RR (95% CI)** | **RR (95% CI)** | **RR (95% CI)** | **RR (95% CI)** | **RR (95% CI)** | **RR (95% CI)** |  |
| **Age** | | | | | | | | | | | | | | | |
| 15-19 | 0.11 (0.10, 0.12) | 0.11 (0.11, 0.12) | 0.05 (0.05, 0.05) | 0.05 (0.05, 0.05) | 0.07 (0.06, 0.07) | 0.07 (0.06, 0.07) | 0.12 (0.12, 0.12) | 0.12 (0.11, 0.12) | 0.08 (0.07, 0.09) | 0.09 (0.09, 0.10) | 0.04 (0.04, 0.04) | 0.04 (0.03, 0.04) | 0.07 (0.07, 0.07) | 0.07 (0.07, 0.07) | |
| 20-24 | 0.15 (0.14, 0.17) | 0.17 (0.16, 0.18) | 0.09 (0.09, 0.09) | 0.10 (0.09, 0.10) | 0.09 (0.09, 0.10) | 0.12 (0.11, 0.13) | 0.18 (0.17, 0.18) | 0.18 (0.17, 0.18) | 0.14 (0.13, 0.15) | 0.16 (0.15, 0.17) | 0.07 (0.07, 0.07) | 0.08 (0.07, 0.08) | 0.11 (0.11, 0.12) | 0.13 (0.13, 0.13) | |
| 25-29 | 0.23 (0.22, 0.25) | 0.24 (0.23, 0.25) | 0.14 (0.13, 0.14) | 0.17 (0.16, 0.18) | 0.17 (0.15, 0.18) | 0.21 (0.19, 0.22) | 0.24 (0.24, 0.25) | 0.23 (0.22, 0.23) | 0.26 (0.24, 0.27) | 0.30 (0.28, 0.31) | 0.14 (0.13, 0.14) | 0.15 (0.15, 0.15) | 0.18 (0.17, 0.18) | 0.18 (0.18, 0.19) | |
| 30-34 | 0.41 (0.38, 0.44) | 0.39 (0.37, 0.41) | 0.26 (0.25, 0.27) | 0.31 (0.30, 0.32) | 0.28 (0.25, 0.30) | 0.36 (0.33, 0.39) | 0.40 (0.39, 0.41) | 0.37 (0.36, 0.38) | 0.45 (0.42, 0.48) | 0.46 (0.43, 0.48) | 0.26 (0.25, 0.27) | 0.29 (0.28, 0.30) | 0.31 (0.31, 0.32) | 0.31 (0.30, 0.31) | |
| 35-39 | 0.59 (0.55, 0.63) | 0.55 (0.52, 0.58) | 0.40 (0.39, 0.41) | 0.50 (0.48, 0.52) | 0.38 (0.35, 0.42) | 0.51 (0.47, 0.55) | 0.57 (0.56, 0.59) | 0.55 (0.53, 0.56) | 0.61 (0.58, 0.66) | 0.65 (0.61, 0.68) | 0.45 (0.44, 0.46) | 0.49 (0.48, 0.51) | 0.46 (0.45, 0.47) | 0.45 (0.45, 0.46) | |
| 40-44 | 0.76 (0.71, 0.82) | 0.72 (0.68, 0.76) | 0.63 (0.62, 0.65) | 0.76 (0.73, 0.80) | 0.56 (0.51, 0.61) | 0.73 (0.67, 0.79) | 0.79 (0.77, 0.81) | 0.81 (0.78, 0.83) | 0.87 (0.82, 0.93) | 0.96 (0.91, 1.01) | 0.77 (0.75, 0.80) | 0.83 (0.81, 0.86) | 0.65 (0.63, 0.66) | 0.66 (0.65, 0.67) | |
| 45-49 | 0.96 (0.89, 1.03) | 0.82 (0.77, 0.87) | 0.99 (0.97, 1.02) | 1.13 (1.08, 1.18) | 0.81 (0.74, 0.89) | 1.02 (0.94, 1.10) | 1.21 (1.18, 1.25) | 1.16 (1.13, 1.19) | 1.27 (1.19, 1.36) | 1.31 (1.24, 1.39) | 1.32 (1.28, 1.36) | 1.34 (1.30, 1.37) | 0.95 (0.92, 0.97) | 0.96 (0.94, 0.97) | |
| 50-54 | 1.21 (1.12, 1.31) | 1.17 (1.10, 1.23) | 1.53 (1.49, 1.58) | 1.53 (1.47, 1.60) | 1.18 (1.07, 1.30) | 1.24 (1.14, 1.34) | 1.58 (1.54, 1.63) | 1.56 (1.52, 1.61) | 1.79 (1.67, 1.92) | 1.71 (1.62, 1.81) | 2.02 (1.96, 2.08) | 1.86 (1.81, 1.91) | 1.36 (1.32, 1.40) | 1.35 (1.33, 1.37) | |
| 55-59 | 1.48 (1.37, 1.60) | 1.45 (1.37, 1.54) | 2.13 (2.07, 2.19) | 1.82 (1.74, 1.90) | 1.56 (1.42, 1.72) | 1.41 (1.30, 1.53) | 1.97 (1.91, 2.03) | 1.89 (1.84, 1.94) | 2.28 (2.13, 2.44) | 2.04 (1.93, 2.16) | 2.65 (2.57, 2.73) | 2.27 (2.20, 2.33) | 1.82 (1.77, 1.87) | 1.67 (1.64, 1.70) | |
| 60-64 | 1.65 (1.53, 1.79) | 1.66 (1.57, 1.76) | 2.68 (2.61, 2.76) | 2.00 (1.92, 2.09) | 1.98 (1.80, 2.18) | 1.51 (1.39, 1.65) | 2.29 (2.22, 2.35) | 2.17 (2.12, 2.23) | 2.75 (2.57, 2.95) | 2.50 (2.37, 2.65) | 3.13 (3.04, 3.22) | 2.55 (2.48, 2.62) | 2.26 (2.19, 2.32) | 1.93 (1.90, 1.96) | |
| 65-69 | 1.87 (1.73, 2.02) | 1.93 (1.83, 2.04) | 3.02 (2.94, 3.11) | 2.07 (1.98, 2.16) | 2.30 (2.09, 2.54) | 1.70 (1.56, 1.84) | 2.51 (2.44, 2.58) | 2.40 (2.34, 2.47) | 3.16 (2.96, 3.38) | 2.82 (2.67, 2.98) | 3.29 (3.19, 3.38) | 2.68 (2.61, 2.75) | 2.60 (2.53, 2.67) | 2.17 (2.13, 2.20) | |
| 70-74 | 2.18 (2.02, 2.35) | 2.41 (2.28, 2.55) | 3.14 (3.06, 3.23) | 2.18 (2.09, 2.27) | 2.71 (2.47, 2.97) | 2.01 (1.86, 2.18) | 2.54 (2.47, 2.61) | 2.66 (2.59, 2.73) | 3.02 (2.83, 3.23) | 2.53 (2.39, 2.67) | 3.21 (3.12, 3.31) | 2.73 (2.66, 2.80) | 2.83 (2.75, 2.91) | 2.44 (2.40, 2.48) | |
| 75-79 | 2.36 (2.19, 2.54) | 2.62 (2.49, 2.77) | 3.11 (3.03, 3.20) | 2.36 (2.26, 2.46) | 3.14 (2.87, 3.45) | 2.51 (2.32, 2.72) | 2.42 (2.35, 2.49) | 2.61 (2.54, 2.68) | 2.86 (2.68, 3.05) | 2.57 (2.43, 2.71) | 3.03 (2.95, 3.12) | 2.84 (2.77, 2.92) | 2.92 (2.85, 3.00) | 2.65 (2.61, 2.69) | |
| 80-84 | 2.32 (2.15, 2.49) | 2.65 (2.51, 2.80) | 3.11 (3.03, 3.19) | 2.63 (2.53, 2.74) | 3.51 (3.21, 3.85) | 3.07 (2.84, 3.32) | 2.03 (1.98, 2.09) | 2.29 (2.23, 2.35) | 2.48 (2.33, 2.65) | 2.02 (1.92, 2.13) | 2.75 (2.68, 2.83) | 2.76 (2.69, 2.83) | 2.91 (2.84, 2.99) | 2.79 (2.75, 2.83) | |
| 85-89 | 3.10 (2.88, 3.33) | 2.61 (2.47, 2.75) | 3.29 (3.21, 3.38) | 3.24 (3.11, 3.38) | 3.61 (3.29, 3.95) | 3.10 (2.87, 3.35) | 1.68 (1.63, 1.73) | 1.92 (1.87, 1.96) | 1.30 (1.22, 1.38) | 1.25 (1.18, 1.32) | 2.56 (2.49, 2.63) | 2.81 (2.73, 2.88) | 3.00 (2.92, 3.08) | 2.96 (2.92, 3.01) | |
| 90-94 | 3.73 (3.46, 4.02) | 2.58 (2.45, 2.73) | 3.45 (3.36, 3.55) | 4.14 (3.97, 4.32) | 3.84 (3.49, 4.21) | 3.67 (3.39, 3.97) | 1.56 (1.52, 1.60) | 1.62 (1.58, 1.67) | 1.09 (1.02, 1.16) | 1.33 (1.26, 1.41) | 2.51 (2.44, 2.58) | 2.93 (2.86, 3.01) | 3.03 (2.95, 3.11) | 3.45 (3.40, 3.51) | |
| 95+ | 1.92 (1.77, 2.08) | 2.77 (2.61, 2.94) | 2.85 (2.76, 2.93) | 4.21 (4.02, 4.41) | 5.90 (5.33, 6.53) | 5.81 (5.33, 6.34) | 1.51 (1.46, 1.55) | 1.49 (1.45, 1.54) | 1.03 (0.96, 1.10) | 1.15 (1.08, 1.22) | 2.38 (2.31, 2.45) | 3.04 (2.95, 3.13) | 2.51 (2.44, 2.58) | 3.73 (3.66, 3.79) | |
| **Period** | | | | | | | | | | | | | | | |
| 1992-1996 | 0.82 (0.79, 0.86) | 1.03 (1.00, 1.06) | 0.83 (0.81, 0.84) | 0.85 (0.83, 0.86) | 0.87 (0.83, 0.91) | 0.95 (0.91, 0.99) | 0.84 (0.83, 0.85) | 0.85 (0.84, 0.87) | 0.79 (0.77, 0.82) | 0.91 (0.88, 0.93) | 0.85 (0.84, 0.86) | 0.84 (0.83, 0.85) | 0.83 (0.82, 0.84) | 0.91 (0.90, 0.91) | |
| 1997-2001 | 0.86 (0.83, 0.90) | 1.03 (1.00, 1.06) | 0.88 (0.87, 0.90) | 0.88 (0.87, 0.90) | 0.93 (0.88, 0.98) | 0.95 (0.91, 0.99) | 0.89 (0.87, 0.90) | 0.90 (0.89, 0.92) | 0.84 (0.81, 0.87) | 0.94 (0.92, 0.97) | 0.91 (0.89, 0.92) | 0.92 (0.91, 0.93) | 0.88 (0.87, 0.90) | 0.94 (0.94, 0.95) | |
| 2002-2006 | 0.93 (0.90, 0.97) | 0.98 (0.96, 1.01) | 0.94 (0.93, 0.96) | 0.94 (0.92, 0.97) | 0.96 (0.92, 1.01) | 0.97 (0.93, 1.01) | 0.95 (0.94, 0.97) | 0.96 (0.95, 0.97) | 0.94 (0.90, 0.97) | 0.92 (0.89, 0.94) | 0.97 (0.96, 0.99) | 0.98 (0.97, 0.99) | 0.95 (0.94, 0.96) | 0.97 (0.96, 0.97) | |
| 2007-2011 | 1.00 (0.96, 1.04) | 0.94 (0.91, 0.97) | 1.02 (1.01, 1.03) | 1.00 (0.98, 1.02) | 1.04 (0.99, 1.09) | 1.03 (0.99, 1.07) | 1.06 (1.04, 1.07) | 1.06 (1.04, 1.07) | 1.04 (1.00, 1.07) | 0.99 (0.96, 1.02) | 1.02 (1.01, 1.04) | 1.02 (1.01, 1.04) | 1.02 (1.00, 1.03) | 0.99 (0.99, 1.00) | |
| 2012-2016 | 1.13 (1.09, 1.17) | 0.95 (0.92, 0.98) | 1.16 (1.14, 1.17) | 1.14 (1.12, 1.17) | 1.07 (1.02, 1.13) | 1.02 (0.98, 1.07) | 1.12 (1.11, 1.14) | 1.10 (1.09, 1.12) | 1.17 (1.13, 1.21) | 1.10 (1.07, 1.13) | 1.10 (1.09, 1.12) | 1.10 (1.08, 1.11) | 1.12 (1.11, 1.14) | 1.06 (1.05, 1.06) | |
| 2017-2021 | 1.34 (1.28, 1.39) | 1.07 (1.04, 1.11) | 1.23 (1.21, 1.25) | 1.24 (1.21, 1.27) | 1.15 (1.10, 1.21) | 1.10 (1.05, 1.15) | 1.18 (1.16, 1.20) | 1.16 (1.14, 1.17) | 1.32 (1.28, 1.37) | 1.17 (1.13, 1.20) | 1.18 (1.16, 1.20) | 1.17 (1.16, 1.19) | 1.26 (1.24, 1.28) | 1.16 (1.15, 1.17) | |
| **Cohort** | | | | | | | | | | | | | | | |
| 1897-1901 | 2.42 (2.03, 2.87) | 2.45 (2.16, 2.77) | 3.03 (2.84, 3.22) | 2.10 (1.91, 2.32) | 2.75 (2.21, 3.41) | 2.20 (1.83, 2.64) | 2.46 (2.30, 2.62) | 2.37 (2.23, 2.52) | 2.23 (1.91, 2.59) | 1.63 (1.43, 1.85) | 4.08 (3.82, 4.36) | 3.80 (3.57, 4.04) | 3.00 (2.82, 3.20) | 2.72 (2.62, 2.82) | |
| 1902-1906 | 2.17 (1.92, 2.46) | 2.22 (2.03, 2.43) | 2.83 (2.71, 2.96) | 2.26 (2.11, 2.42) | 2.47 (2.12, 2.88) | 1.92 (1.69, 2.19) | 2.24 (2.14, 2.35) | 2.24 (2.15, 2.34) | 2.32 (2.08, 2.59) | 1.71 (1.56, 1.87) | 3.68 (3.51, 3.86) | 3.48 (3.33, 3.63) | 2.64 (2.53, 2.76) | 2.54 (2.48, 2.61) | |
| 1907-1911 | 2.06 (1.86, 2.28) | 1.99 (1.85, 2.15) | 2.79 (2.69, 2.90) | 2.43 (2.30, 2.57) | 2.27 (2.00, 2.58) | 1.87 (1.68, 2.08) | 2.01 (1.93, 2.08) | 2.05 (1.98, 2.12) | 2.08 (1.91, 2.28) | 1.72 (1.60, 1.86) | 3.27 (3.15, 3.40) | 3.14 (3.03, 3.25) | 2.43 (2.34, 2.52) | 2.39 (2.34, 2.44) | |
| 1912-1916 | 2.01 (1.84, 2.20) | 1.78 (1.67, 1.90) | 2.64 (2.56, 2.73) | 2.57 (2.45, 2.70) | 2.12 (1.90, 2.36) | 2.03 (1.85, 2.23) | 1.81 (1.75, 1.87) | 1.87 (1.81, 1.93) | 1.80 (1.67, 1.95) | 1.66 (1.55, 1.77) | 2.85 (2.76, 2.95) | 2.78 (2.70, 2.87) | 2.29 (2.22, 2.37) | 2.31 (2.26, 2.35) | |
| 1917-1921 | 1.94 (1.80, 2.11) | 1.65 (1.56, 1.75) | 2.35 (2.2 8, 2.42) | 2.51 (2.40, 2.62) | 2.01 (1.82, 2.22) | 2.16 (1.99, 2.35) | 1.65 (1.60, 1.70) | 1.73 (1.69, 1.78) | 1.58 (1.47, 1.70) | 1.64 (1.55, 1.74) | 2.41 (2.33, 2.48) | 2.41 (2.34, 2.48) | 2.05 (2.00, 2.11) | 2.08 (2.05, 2.12) | |
| 1922-1926 | 1.77 (1.65, 1.91) | 1.63 (1.54, 1.72) | 2.02 (1.97, 2.07) | 2.25 (2.16, 2.35) | 2.00 (1.83, 2.19) | 2.18 (2.02, 2.36) | 1.52 (1.47, 1.56) | 1.60 (1.56, 1.64) | 1.54 (1.44, 1.64) | 1.62 (1.54, 1.71) | 2.02 (1.96, 2.07) | 2.05 (2.00, 2.11) | 1.95 (1.90, 2.00) | 1.96 (1.93, 1.99) | |
| 1927-1931 | 1.61 (1.50, 1.74) | 1.62 (1.53, 1.72) | 1.91 (1.86, 1.97) | 1.98 (1.90, 2.07) | 2.00 (1.82, 2.20) | 2.19 (2.02, 2.37) | 1.39 (1.35, 1.43) | 1.47 (1.43, 1.51) | 1.49 (1.39, 1.59) | 1.61 (1.52, 1.70) | 1.69 (1.64, 1.74) | 1.75 (1.71, 1.80) | 1.78 (1.73, 1.83) | 1.77 (1.74, 1.80) | |
| 1932-1936 | 1.54 (1.42, 1.67) | 1.58 (1.49, 1.67) | 1.72 (1.67, 1.77) | 1.73 (1.66, 1.81) | 1.82 (1.65, 2.01) | 1.96 (1.81, 2.14) | 1.28 (1.25, 1.32) | 1.35 (1.32, 1.39) | 1.44 (1.34, 1.54) | 1.60 (1.51, 1.69) | 1.39 (1.35, 1.44) | 1.48 (1.44, 1.52) | 1.58 (1.54, 1.62) | 1.57 (1.54, 1.59) | |
| 1937-1941 | 1.43 (1.32, 1.55) | 1.50 (1.41, 1.59) | 1.48 (1.44, 1.53) | 1.52 (1.45, 1.59) | 1.75 (1.58, 1.94) | 1.74 (1.60, 1.90) | 1.19 (1.16, 1.23) | 1.25 (1.21, 1.28) | 1.25 (1.17, 1.34) | 1.48 (1.40, 1.57) | 1.13 (1.10, 1.17) | 1.21 (1.17, 1.24) | 1.39 (1.35, 1.43) | 1.39 (1.37, 1.41) | |
| 1942-1946 | 1.29 (1.19, 1.40) | 1.39 (1.31, 1.48) | 1.31 (1.27, 1.35) | 1.32 (1.26, 1.39) | 1.64 (1.48, 1.82) | 1.57 (1.44, 1.72) | 1.12 (1.08, 1.15) | 1.16 (1.12, 1.19) | 1.13 (1.05, 1.21) | 1.40 (1.32, 1.49) | 0.93 (0.90, 0.96) | 0.98 (0.96, 1.01) | 1.22 (1.19, 1.26) | 1.23 (1.21, 1.26) | |
| 1947-1951 | 1.19 (1.09, 1.29) | 1.28 (1.21, 1.36) | 1.16 (1.13, 1.20) | 1.18 (1.12, 1.23) | 1.37 (1.23, 1.52) | 1.24 (1.14, 1.36) | 1.04 (1.01, 1.08) | 1.08 (1.05, 1.11) | 1.04 (0.97, 1.12) | 1.28 (1.20, 1.36) | 0.78 (0.76, 0.81) | 0.82 (0.79, 0.84) | 1.10 (1.06, 1.13) | 1.10 (1.08, 1.12) | |
| 1952-1956 | 1.06 (0.97, 1.15) | 1.15 (1.08, 1.22) | 1.08 (1.05, 1.11) | 1.09 (1.04, 1.14) | 1.27 (1.14, 1.41) | 1.05 (0.96, 1.15) | 0.97 (0.94, 1.00) | 0.99 (0.96, 1.02) | 0.96 (0.89, 1.03) | 1.14 (1.07, 1.21) | 0.73 (0.71, 0.75) | 0.73 (0.71, 0.75) | 1.00 (0.97, 1.03) | 1.00 (0.99, 1.02) | |
| 1957-1961 | 0.94 (0.87, 1.02) | 1.02 (0.96, 1.08) | 0.89 (0.86, 0.92) | 0.90 (0.86, 0.94) | 1.13 (1.02, 1.25) | 0.91 (0.84, 1.00) | 0.90 (0.88, 0.93) | 0.91 (0.89, 0.94) | 0.86 (0.80, 0.92) | 0.95 (0.90, 1.01) | 0.68 (0.66, 0.70) | 0.69 (0.67, 0.71) | 0.88 (0.86, 0.91) | 0.89 (0.88, 0.91) | |
| 1962-1966 | 0.82 (0.76, 0.89) | 0.87 (0.82, 0.92) | 0.74 (0.72, 0.77) | 0.77 (0.74, 0.81) | 1.02 (0.92, 1.13) | 0.85 (0.78, 0.93) | 0.84 (0.82, 0.87) | 0.84 (0.82, 0.87) | 0.79 (0.73, 0.84) | 0.86 (0.81, 0.91) | 0.65 (0.63, 0.67) | 0.65 (0.64, 0.67) | 0.78 (0.76, 0.80) | 0.79 (0.78, 0.80) | |
| 1967-1971 | 0.76 (0.70, 0.82) | 0.78 (0.73, 0.82) | 0.65 (0.63, 0.67) | 0.67 (0.64, 0.70) | 0.83 (0.75, 0.91) | 0.73 (0.68, 0.80) | 0.78 (0.75, 0.80) | 0.77 (0.75, 0.79) | 0.75 (0.70, 0.80) | 0.77 (0.72, 0.81) | 0.60 (0.59, 0.62) | 0.62 (0.60, 0.63) | 0.72 (0.70, 0.74) | 0.71 (0.70, 0.72) | |
| 1972-1976 | 0.68 (0.63, 0.73) | 0.68 (0.64, 0.72) | 0.56 (0.55, 0.58) | 0.58 (0.56, 0.61) | 0.68 (0.62, 0.74) | 0.65 (0.60, 0.71) | 0.72 (0.70, 0.74) | 0.71 (0.69, 0.73) | 0.71 (0.67, 0.76) | 0.69 (0.65, 0.73) | 0.58 (0.56, 0.60) | 0.58 (0.56, 0.59) | 0.64 (0.62, 0.66) | 0.63 (0.62, 0.64) | |
| 1977-1981 | 0.64 (0.60, 0.69) | 0.61 (0.58, 0.64) | 0.51 (0.49, 0.52) | 0.52 (0.50, 0.54) | 0.55 (0.50, 0.60) | 0.58 (0.54, 0.63) | 0.66 (0.64, 0.67) | 0.65 (0.63, 0.66) | 0.65 (0.61, 0.70) | 0.63 (0.59, 0.66) | 0.58 (0.56, 0.59) | 0.57 (0.56, 0.59) | 0.56 (0.55, 0.58) | 0.56 (0.55, 0.57) | |
| 1982-1986 | 0.57 (0.52, 0.61) | 0.53 (0.50, 0.56) | 0.44 (0.43, 0.46) | 0.46 (0.44, 0.48) | 0.42 (0.38, 0.46) | 0.49 (0.45, 0.53) | 0.60 (0.58, 0.62) | 0.59 (0.57, 0.60) | 0.56 (0.53, 0.60) | 0.52 (0.49, 0.55) | 0.55 (0.54, 0.57) | 0.55 (0.53, 0.56) | 0.49 (0.48, 0.51) | 0.49 (0.48, 0.50) | |
| 1987-1991 | 0.48 (0.44, 0.52) | 0.45 (0.42, 0.48) | 0.39 (0.37, 0.40) | 0.41 (0.39, 0.43) | 0.31 (0.28, 0.35) | 0.38 (0.34, 0.41) | 0.55 (0.54, 0.57) | 0.53 (0.51, 0.55) | 0.50 (0.47, 0.54) | 0.47 (0.44, 0.50) | 0.51 (0.49, 0.53) | 0.50 (0.48, 0.51) | 0.44 (0.43, 0.46) | 0.43 (0.43, 0.44) | |
| 1992-1996 | 0.39 (0.36, 0.43) | 0.39 (0.37, 0.42) | 0.33 (0.32, 0.34) | 0.34 (0.32, 0.36) | 0.25 (0.22, 0.29) | 0.30 (0.27, 0.34) | 0.51 (0.49, 0.53) | 0.48 (0.46, 0.49) | 0.50 (0.46, 0.54) | 0.44 (0.40, 0.47) | 0.43 (0.42, 0.45) | 0.43 (0.42, 0.45) | 0.37 (0.36, 0.38) | 0.37 (0.37, 0.38) | |
| 1997-2001 | 0.33 (0.29, 0.37) | 0.34 (0.31, 0.37) | 0.27 (0.26, 0.28) | 0.28 (0.26, 0.30) | 0.21 (0.18, 0.24) | 0.28 (0.25, 0.32) | 0.46 (0.44, 0.48) | 0.42 (0.40, 0.44) | 0.48 (0.44, 0.54) | 0.46 (0.42, 0.50) | 0.33 (0.31, 0.34) | 0.34 (0.33, 0.36) | 0.31 (0.29, 0.32) | 0.32 (0.31, 0.33) | |
| 2002-2006 | 0.25 (0.21, 0.31) | 0.27 (0.23, 0.31) | 0.22 (0.21, 0.24) | 0.27 (0.24, 0.30) | 0.17 (0.14, 0.22) | 0.25 (0.21, 0.31) | 0.39 (0.36, 0.41) | 0.35 (0.32, 0.37) | 0.44 (0.37, 0.51) | 0.46 (0.40, 0.53) | 0.29 (0.27, 0.31) | 0.26 (0.24, 0.28) | 0.25 (0.23, 0.26) | 0.27 (0.26, 0.28) | |

RR: relative risk; CI: confidence interval; DALYs: disability-adjusted life years.
