# Supplementary material for: Trends in incidence, mortality and disability-adjusted life years of colorectal cancer in East Asia (1990–2021): An analysis of the Global Burden of Disease study 2021
Source: PLoS One. 2025 Oct 8;20(10):e0334229. doi: 10.1371/journal.pone.0334229 (PMC12507298; doi:10.1371/journal.pone.0334229)
Supplement: S7 Table — (DOCX) [file pone.0334229.s007.docx]

**S7 Table.** **Changes in CRC cases, deaths, and DALY numbers according to population-level determinants of ageing, population growth, and epidemiological changes from 1990 to 2021 in five East Asian countries, the United States, and globally for males and females**

| **Countries** | **Male** | | | | | | | | **Female** | | | | | | | | | | | | |  |
| --- | --- | --- | --- | --- | --- | --- | --- | --- | --- | --- | --- | --- | --- | --- | --- | --- | --- | --- | --- | --- | --- | --- |
|  | **Overall difference ^a^** | | **Ageing ^b^** | | **Population ^c^** | | **Epidemiological change ^d^** | | **Overall difference ^a^** | | **Ageing ^b^** | | **Population ^c^** | | | | | **Epidemiological change ^d^** | | | |  |
|  | **Number** | **Percentage, %** | **Number** | **Percentage, %** | **Number** | **Percentage, %** | **Number** | **Percentage, %** | **Number** | **Percentage, %** | **Number** | **Percentage, %** | | **Number** | | **Percentage, %** | | | **Number** | | **Percentage, %** | |
| **Cases** | | | | | | | | | | | | | | | | | | | | | |  |
| China | 330642.36 | 374.16 | 130338.80 | 147.49 | 64018.32 | 72.44 | 136285.15 | 154.22 | 169289.70 | 241.78 | 84246.91 | 120.32 | | | 44263.01 | | 63.22 | | | 40779.77 | 58.24 | |
| Japan | 57776.80 | 140.33 | 42487.36 | 103.19 | 5530.15 | 13.43 | 9759.29 | 23.70 | 40177.00 | 125.88 | 31433.10 | 98.49 | | | 4784.06 | | 14.99 | | | 3959.84 | 12.41 | |
| South Korea | 17255.33 | 587.50 | 8295.14 | 282.43 | 3141.61 | 106.96 | 5818.58 | 198.11 | 10255.37 | 380.26 | 5173.79 | 191.84 | | | 2115.65 | | 78.45 | | | 2965.93 | 109.97 | |
| North Korea | 1633.55 | 146.61 | 505.00 | 45.32 | 843.10 | 75.67 | 285.41 | 25.61 | 1257.97 | 110.12 | 588.52 | 51.52 | | | 539.91 | | 47.26 | | | 129.54 | 11.34 | |
| Mongolia | 79.91 | 189.77 | 15.15 | 35.98 | 41.55 | 98.68 | 23.20 | 55.10 | 68.79 | 144.98 | 18.71 | 39.44 | | | 46.14 | | 97.25 | | | 3.93 | 8.29 | |
| United States | 38370.32 | 49.74 | 33223.13 | 43.07 | 32169.94 | 41.70 | −27022.75 | −35.03 | 23415.68 | 31.14 | 18708.80 | 24.88 | | | 27101.95 | | 36.05 | | | −22395.07 | −29.79 | |
| Global | 793699.75 | 168.96 | 273630.83 | 58.25 | 388822.01 | 82.77 | 131246.91 | 27.94 | 483859.97 | 108.29 | 198517.44 | 44.43 | | | 326058.84 | | 72.97 | | | −40716.31 | −9.11% | |
| **Deaths** | | | | | | | | | | | | | | | | | | | | | | |
| China | 108164.45 | 163.30 | 75552.74 | 114.07 | 33127.59 | 50.01 | −515.88 | −0.78 | 47661.28 | 89.81 | 51620.45 | 97.27 | | | 24440.41 | | 46.05 | | | −28399.58 | −53.52 | |
| Japan | 18022.31 | 107.36 | 20243.24 | 120.59 | 2113.10 | 12.59 | −4334.02 | −25.82 | 18549.98 | 127.37 | 19565.22 | 134.34 | | | 2222.19 | | 15.26 | | | −3237.43 | −22.23 | |
| South Korea | 4603.70 | 240.37 | 3888.41 | 203.02 | 1315.65 | 68.69 | −600.36 | −31.35 | 3256.19 | 172.73 | 2992.02 | 1059.28 | | | 1059.28 | | 56.19 | | | −795.12 | −42.18 | |
| North Korea | 895.95 | 104.30 | 394.21 | 45.89 | 588.23 | 68.48 | −86.49 | −10.07 | 734.48 | 79.59 | 515.64 | 55.88 | | | 402.78 | | 43.65 | | | −183.94 | −19.93 | |
| Mongolia | 52.99 | 139.36 | 10.84 | 28.52 | 33.69 | 88.61 | 8.45 | 22.22 | 45.48 | 105.74 | 13.37 | 31.09 | | | 38.27 | | 88.99 | | | −6.17 | −14.34 | |
| United States | 6321.84 | 19.13 | 14135.27 | 42.76 | 12522.69 | 37.89 | −20336.13 | −61.52 | 1533.54 | 4.49 | 8155.67 | 23.86 | | | 11182.61 | | 32.72 | | | −17804.74 | −52.10 | |
| Global | 293846.59 | 102.13 | 157129.78 | 54.61 | 204664.08 | 71.14 | −67947.27 | −23.62 | 179907.07 | 63.66 | 124585.05 | 44.08 | | | 183966.79 | | 65.10 | | | −128644.77 | −45.52 | |
| **DALYs** | | | | | | | | | | | | | | | | | | | | | | |
| China | 2448410.16 | 120.03 | 1596514.98 | 78.27 | 910765.77 | 44.65 | −58870.59 | −2.89 | 834784.03 | 54.73 | 1055242.88 | 69.18 | | | 624764.57 | | 40.96 | | | −845223.43 | −55.41 | |
| Japan | 241717.85 | 57.24 | 297274.44 | 70.39 | 44416.28 | 10.52 | −99972.87 | −23.67 | 177676.27 | 53.86 | 228089.37 | 69.14 | | | 39616.72 | | 12.01 | | | −90029.82 | −27.29 | |
| South Korea | 90800.90 | 159.22 | 78359.98 | 137.41 | 32632.93 | 57.22 | −20192.01 | −35.41 | 45069.87 | 86.66 | 49765.02 | 95.69 | | | 23360.94 | | 44.92 | | | −28056.10 | −53.95 | |
| North Korea | 25690.41 | 92.20 | 9141.84 | 32.81 | 18405.81 | 66.06 | −1857.24 | −6.67 | 15661.32 | 60.50 | 9408.12 | 36.34 | | | 10573.40 | | 40.84 | | | −4320.21 | −16.69 | |
| Mongolia | 1723.15 | 152.64 | 428.72 | 37.98 | 1029.87 | 91.23 | 264.56 | 23.43 | 1298.46 | 103.82 | 514.68 | 41.15 | | | 1109.77 | | 88.74 | | | −325.99 | −26.07 | |
| United States | 177637.38 | 23.21 | 281184.68 | 36.74 | 292448.58 | 38.22 | −395995.88 | −51.75 | 52858.54 | 7.59 | 159521.99 | 22.09 | | | 230830.08 | | 33.13 | | | −337493.54 | −48.45 | |
| Global | 6557546.51 | 86.17 | 3075855.75 | 40.42 | 5167553.79 | 67.91 | −1685863.03 | −22.15 | 3446895.95 | 50.79 | 2251179.60 | 33.17 | | | 4233591.48 | | 62.38 | | | −3037875.12 | −44.76 | |

^a^ Overall difference, the overall change contributed by all three components from 1990 to 2021.

^b^ Ageing, change due to change in the age structure.

^c^ Population, change due to change in population number.

^d^ Epidemiological change, change due to epidemiologic changes. Epidemiologic changes refer to the number change when age structure and population hold constant.

DALYs: disability-adjusted life years.
