# Supplementary material for: Trends in incidence, mortality and disability-adjusted life years of colorectal cancer in East Asia (1990–2021): An analysis of the Global Burden of Disease study 2021
Source: PLoS One. 2025 Oct 8;20(10):e0334229. doi: 10.1371/journal.pone.0334229 (PMC12507298; doi:10.1371/journal.pone.0334229)
Supplement: S8 Table — (DOCX) [file pone.0334229.s008.docx]

**S8 Table. Spearman’s rank correlation coefficient between age-standardised CRC burden and SDI across five East Asian countries, the United States, and globally from 1990 to 2021**

| **Countries** | **Age-standardised incidence rate** | | | | **Age-standardised mortality rate** | | | | **Age-standardised DALY rate** | | | |
| --- | --- | --- | --- | --- | --- | --- | --- | --- | --- | --- | --- | --- |
|  | **Male** | | **Female** | | **Male** | | **Female** | | **Male** | | **Female** | |
|  | **r** | **p value** | **r** | **p value** | **r** | **p value** | **r** | **p value** | **r** | **p value** | **r** | **p value** |
| China | 1.00 | < 0.001 | 0.99 | < 0.001 | 0.58 | < 0.001 | -0.96 | < 0.001 | 0.12, | 0.525 | -0.96, | < 0.001 |
| Japan | 0.66 | < 0.001 | 0.47 | < 0.01 | -0.97 | < 0.001 | -0.95 | < 0.001 | -0.97, | < 0.001 | -0.97, | < 0.001 |
| South Korea | 0.82 | < 0.001 | 0.74 | < 0.001 | -0.51 | < 0.01 | -0.91 | < 0.001 | -0.64, | < 0.001 | -0.99, | < 0.001 |
| North Korea | 0.87 | < 0.001 | 0.77 | < 0.001 | -0.11 | 0.546 | -0.54 | < 0.01 | -0.03, | 0.880 | -0.67, | < 0.001 |
| Mongolia | 0.95 | < 0.001 | 0.22 | 0.215 | 0.78 | < 0.001 | -0.58 | < 0.001 | 0.73, | < 0.001 | -0.81, | < 0.001 |
| United States | -0.91 | < 0.001 | -0.86 | < 0.001 | -1.00 | < 0.001 | -1.00 | < 0.001 | -1.00, | < 0.001 | -1.00, | < 0.001 |
| Global | 1.00 | < 0.001 | -0.93 | < 0.001 | -1.00 | < 0.001 | -1.00 | < 0.001 | -1.00, | < 0.001 | -1.00, | < 0.001 |

SDI: socio-demographic index; DALY: disability-adjusted life year.
